# Targeting hyaluronic acid synthase-3 (HAS3) for the treatment of advanced renal cell carcinoma

**DOI:** 10.1186/s12935-022-02818-1

**Published:** 2022-12-29

**Authors:** Jiaojiao Wang, Andre R. Jordan, Huabin Zhu, Sarrah L. Hasanali, Eric Thomas, Soum D. Lokeshwar, Daley S. Morera, Sung Alexander, Joseph McDaniels, Anuj Sharma, Karina Aguilar, Semih Sarcan, Tianyi Zhu, Mark S. Soloway, Martha K. Terris, Muthusamy Thangaraju, Luis E. Lopez, Vinata B. Lokeshwar

**Affiliations:** 1 Department of Biochemistry and Molecular Biology, Medical College of Georgia, Augusta University, 1410 Laney Walker Blvd, Room CN 1177A, Augusta, GA 30912 USA; 2 Division of Urology, Department of Surgery, Medical College of Georgia, Augusta University, 1410 Laney Walker Blvd, Augusta, GA 30912 USA; 3 Greenbrier High School, Evans, GA 30809 USA; 4 Memorial Healthcare System, Aventura, FL 33180 USA; 5 Present Address: Maoming People’s Hospital, Maoming, China; 6 Present Address: Tulane University School of Medicine, New Orleans, USA; 7 Present Address: Advanced RNA Technologies, Boulder, USA; 8 Present Address: Yale University School of Medicine, New Haven, USA; 9 Present Address: Houston Methodist Hospital, Houston, USA

**Keywords:** HAS3, Hyaluronic acid, 4-methylumbelliferone, Renal cell carcinoma, Molecular targeting, Sorafenib

## Abstract

**Background:**

Hyaluronic acid (HA) promotes cancer metastasis; however, the currently approved treatments do not target HA. Metastatic renal carcinoma (mRCC) is an incurable disease. Sorafenib (SF) is a modestly effective antiangiogenic drug for mRCC. Although only endothelial cells express known SF targets, SF is cytotoxic to RCC cells at concentrations higher than the pharmacological-dose (5-µM). Using patient cohorts, mRCC models, and SF combination with 4-methylumbelliferone (MU), we discovered an SF target in RCC cells and targeted it for treatment.

**Methods:**

We analyzed HA-synthase (HAS1, HAS2, HAS3) expression in RCC cells and clinical (n = 129), TCGA-KIRC (n = 542), and TCGA-KIRP (n = 291) cohorts. We evaluated the efficacy of SF and SF plus MU combination in RCC cells, HAS3-transfectants, endothelial-RCC co-cultures, and xenografts.

**Results:**

RCC cells showed increased HAS3 expression. In the clinical and TCGA-KIRC/TCGA-KIRP cohorts, higher HAS3 levels predicted metastasis and shorter survival. At > 10-µM dose, SF inhibited HAS3/HA-synthesis and RCC cell growth. However, at ≤ 5-µM dose SF in combination with MU inhibited HAS3/HA synthesis, growth of RCC cells and endothelial-RCC co-cultures, and induced apoptosis. The combination inhibited motility/invasion and an HA-signaling-related invasive-signature. We previously showed that MU inhibits SF inactivation in RCC cells. While HAS3-knockdown transfectants were sensitive to SF, ectopic-HAS3-expression induced resistance to the combination. In RCC models, the combination inhibited tumor growth and metastasis with little toxicity; however, ectopic-HAS3-expressing tumors were resistant.

**Conclusion:**

HAS3 is the first known target of SF in RCC cells. In combination with MU (human equivalent-dose, 0.6–1.1-g/day), SF targets HAS3 and effectively abrogates mRCC.

**Supplementary Information:**

The online version contains supplementary material available at 10.1186/s12935-022-02818-1.

## Background

Renal cell carcinoma (RCC) originates from proximal tubular epithelial cells, with clear cell RCC (ccRCC) as the dominant histologic subtype (> 70%–80%). Papillary and chromophobe account for 80% of the non-ccRCC histologic subtypes. Other renal tumors include a sarcomatoid variant, tumors of the collecting duct (both non-ccRCCs), and benign oncocytoma [1–3]. At initial diagnosis, nearly one-third of patients have metastatic disease (mRCC), and approximately one-third more develop metastasis following surgery [4, 5]. Although patients with mRCC receive one of several approved first-line targeted treatments (e.g., tyrosine kinase inhibitors [TKIs]) or immunotherapy, the median survival of the patients is less than two years [6–9]. Therefore, enhancing the efficacy of approved treatments through targeted combinations could improve the clinical outcome.

Sorafenib (SF), a multi-kinase inhibitor, is now not often used in the treatment of mRCC [6, 7, 10, 11]. It is an antiangiogenic drug, and RCC cells do not express the known targets of SF (VEGF-receptor, PDGF-receptor, and c-KIT); these targets are expressed in endothelial cells [12–14]. However, published studies report cytotoxic effects of SF on RCC cells at concentrations higher than pharmacologically achievable (about 5 µM; at the recommended dose of 400 mg b.i.d.) [15–20]. We have demonstrated that 4-methylumbelliferone (MU; 7-Hydroxy-4-methylcoumerin) improves the efficacy of Sorafenib (SF) against mRCC in preclinical models [21].

MU (or Hymecromone) is available through European pharmacies as an over-the-counter drug (Cantabiline) for treating liver ailments. In clinical trials, MU at doses > 1 g per day (range 1.2–2.1 g/day) showed choleretic and antispasmodic properties. Furthermore, MU has a favorable toxicity profile in mice (NIOSH registry) and patients [22–27]. Mechanistic investigations in the non-cancer systems showed that MU is a competitive inhibitor of hyaluronic acid synthesis [28–30]. Subsequently, MU was also shown to block HA synthesis by downregulating HA synthase expression [31, 32]. In prostate and bladder cancer cells systems we showed that at the IC_50_ (0.4 mM) for HA synthesis inhibition, MU exerted cytotoxic effects on tumor cells [25, 27, 33, 34]. However, we also showed that at low doses (0.1–0.2 mM), MU downregulates UDP-glucuronosyltransferase -1A9 (UGT1-A9). UGT1-A9 is a phase II enzyme that inactivates SF [21]. At low doses, MU does not inhibit HA synthesis [25, 34]. Therefore, by blocking the SF’s inactivation, MU improves the efficacy of SF in RCC models. However, it does not explain why SF has activity against RCC cells in the first place, albeit at high doses because RCC cells do not express SF targets. Intriguingly, we found that the effect of SF + MU could be attenuated by HA, suggesting that SF targets the tumor-promoting effects of HA in RCC cells [35].

A non-sulfated glycosaminoglycan, HA promotes tumor growth and metastasis. Furthermore, small HA fragments promote angiogenesis. Three HA-synthases (HAS1, HAS2, and HAS3) synthesize HA of different molecular mass. HAS3 is the most active HA-synthase; it synthesizes HA polymers of about 1 × 10^5^ Dalton. HA synthesized by HAS3 forms a pericellular glycocalyx. The physical properties of HA include keeping the tissues hydrated and osmotically balanced. However, HA also regulates cell adhesion, migration, proliferation, and other cellular activities by binding to its receptors CD44 and RHAMM. These HA-HA receptor interactions induce intracellular signaling [36]. Work from our laboratory and others has shown that overexpression of HA-synthases promotes tumor growth and metastasis [37–39]. We also showed that the expression of HA-synthases and HA receptors is elevated in RCC specimens and correlates with poor clinical outcome in patients with RCC [40].

In this study, we tested the hypothesis that HA-synthesis, in particular, HAS3, is a novel target of SF in RCC cells. Furthermore, MU potentiates the inhibitory effect of SF on HA synthesis at or below the plasma achievable dose (about 5 µM [15]). To test this hypothesis, we investigated the expression of HA synthases (i.e., HAS3) in clinical specimens and correlated it with the clinical outcome. We performed functional and mechanistic studies in preclinical models to test if HAS3 is the primary target of SF in RCC and if, by targeting HA synthesis, the combination effectively controls mRCC.

## Materials and methods

### Cell lines and reagents

We obtained human RCC, normal kidney, and immortalized human microvessel endothelial cell lines from the American Type Culture Collection. The cells were cultured as described before [21, 35]. Cell lines were authenticated, tested for mycoplasma contamination (Genetica DNA Laboratories Inc.), and used within ≤ 10 subculture passages. Additional file 1: Table S1 describes the reagents, cDNA/shRNA constructs, and PCR primers used in this study.

### Specimens and cohorts

In a clinical cohort consisting of the normal kidney (NK, n = 46) and tumor (n = 83) specimens from patients with RCC, we evaluated HAS3 expression (Additional file 1: Table S2) [21]. These specimens were obtained at the University Of Miami Miller School of Medicine and the specimens and the data were transferred to Augusta University in a deidentified manner. Investigators assaying HAS3 expression in these specimens were blinded to the clinical data until data analysis. TCGA datasets for clear cell RCC (ccRCC) specimens (n = 542; TCGA-KIRC) and papillary RCC specimens (n = 291; TCGA-KIRP) were accessed through UCSC-Xena Browser. Clinical, pathological, and follow-up data (metastasis, overall survival [OS]) on all cohorts were used for the analyses (Additional file 1: Table S2).

### HAS3-overexpression and knock-down

Full-length HAS3 cDNA (GenBank: NM_005329.3; NM_001199280.2; AF232772.1) with FLAG-tag or an empty retroviral expression vector (PQCXIH; EV) were stably expressed in RCC cells (786-O and Caki-1) as described before [21]. For HAS3 knock-down, RCC cells were transduced with one of two retroviral expressing shRNA constructs (shRNA#1; shRNA#2). Non-effective scrambled shRNA cassette in the pRS vector was used as a control (Additional file 1: Table S1).

### Phenotypic readout assays for EV and HAS3 transfectants

EV, HAS3, and shRNA transfectants of RCC cells (786-O and Caki-1) were treated with SF (0–15 µM), MU (0–0.2 mM), or SF and MU combinations. Cell proliferation, clonogenic survival, apoptosis, motility, and invasion assays were carried out as described before [21]. Endothelial cells (HMEC-1 or HULEC-5a) were treated with SF (0, 2.5 µM) plus MU (0–0.2 mM) combination with or without 50 µg/ml HA for 48 h. Cytotoxicity was assayed by MTT (3-(4,5-Dimethylthiazol-2-yl)-2,5-diphenyltetrazolium bromide) assay.

### HA-ELISA-like assay (HA test)

RCC tissue (~ 30 mg) homogenates were prepared as described previously [41]. Forty-eight-hour cultures of the transfectants were incubated in serum-free media. Tissue homogenates and conditioned media (CM) were assayed using an HA-ELISA-like assay (HA test). HA levels were normalized to either total protein or to cell number, as described before [41].

### RT-qPCR and immunoblot assays

RCC transfectants were treated with SF, MU, or their combination for 48–60 h. Total RNA isolated from cells and tissues was subjected to a reverse-transcription quantitative polymerase chain reaction (RT-qPCR) described previously [21, 27]. Tissues were lysed in RIPA buffer (50 mM Tris.HCl, pH7.4, 150 mM NaCl, 2 mM each EDTA and EGTA, 50 mM NaF, 0.1 mM sodium orthovanadate, 1% NP40, 0.1% sodium dodecyl sulfate). Tissue and cell lysates were subjected to immunoblot analyses for the indicated proteins.

### Xenograft studies

Xenograft studies were conducted under a protocol approved by the Institutional Animal Care and Use Committee at Augusta University and as described before [21]. In both subcutaneous and orthotopic models, animals in the vehicle and treatment groups were randomly assigned, including the order of the cages for housing, and different individuals prepared the drugs and treated the mice. The animals were housed in the institutional VAF facility, food and water were supplied ad libitum, and pain management after surgery was performed per the institutional guidelines (sustained release Buprenorphine).

Subcutaneous xenograft: As described before [21], athymic mice of both sexes (5–6-week-old) were implanted subcutaneously with a Caki-1 transfectant (EV or HAS3) on the dorsal flanks, together with Matrigel™; one site per mouse. Once the tumor volume was ~ 100 mm^3^, mice received a daily gavage of either vehicle (Kollipore/ ethanol (12.5% final) in 2% sucrose) or SF (30 mg/kg) plus MU (100 or 200 mg/kg) combination prepared in the vehicle. The experimental endpoint was when tumor volume in the vehicle group reached ~ 1000 mm^3^.

Orthotopic model: In athymic mice, we implanted Caki-1-luc (luciferase-expressing) transfectants (EV, HAS3) underneath the renal capsule, as described before [21]. Mice were imaged weekly to determine tumor growth and metastasis. Mice were gavaged daily with either vehicle or SF (30 mg/kg) plus MU (200 mg/kg) combination, starting from day 9. AMIView Software was used to quantify tumor growth.

Histology and immunohistochemistry (IHC): Histology was performed to evaluate tumor cells, metastasis, and organ toxicity. Tissues were analyzed by IHC for HAS3, CD31 (microvessel density), Ki67 (proliferation index), and apoptosis (cleaved caspase 3) as described before [21].

### Sample size calculation

To detect the mean difference in HAS3 levels among tumors for patients who developed mRCC versus those who did not (i.e., 6.5), we needed only 38 tissues for 97% power, type I error 0.05. The sample size in the clinical (n = 129) and TCGA cohorts (KIRC, n = 542; KIRP, n = 291) far exceeded this estimate. In xenograft models, we needed only three mice per group to detect the observed 70% growth inhibition in the combination treatment group, as compared to the vehicle group for 80% power, type I error 0.05. We used five to six mice per group. Therefore, the clinical and preclinical studies were sufficiently powered.

### Statistical analyses

Statistical analyses in this study were performed using JMP Pro 14 and GraphPad Prism 8.0.0 software. HAS3 expression data in the clinical and TCGA cohorts showed a non-normal (asymmetric) distribution. Therefore, we used one-way ANOVA followed by the Mann–Whitney U-test to determine the differences in HAS3 levels among the normal kidney and RCC groups. Univariate logistic regression, Cox proportional hazard (multivariate) model, and Kaplan–Meier plots were used to determine the prognostic capability of HAS3 for predicting the clinical outcome. For rigor, the efficacy of the SF plus MU combination was evaluated in two cell lines and subcutaneous and orthotopic xenograft. Results are expressed as Mean ± SD (or SEM), and one-way ANOVA and unpaired t-test evaluated the significance of the comparisons; all P-values are two-tailed.

## Results

### HAS3 is a potential target of SF in RCC cells

**Fig. 1:**
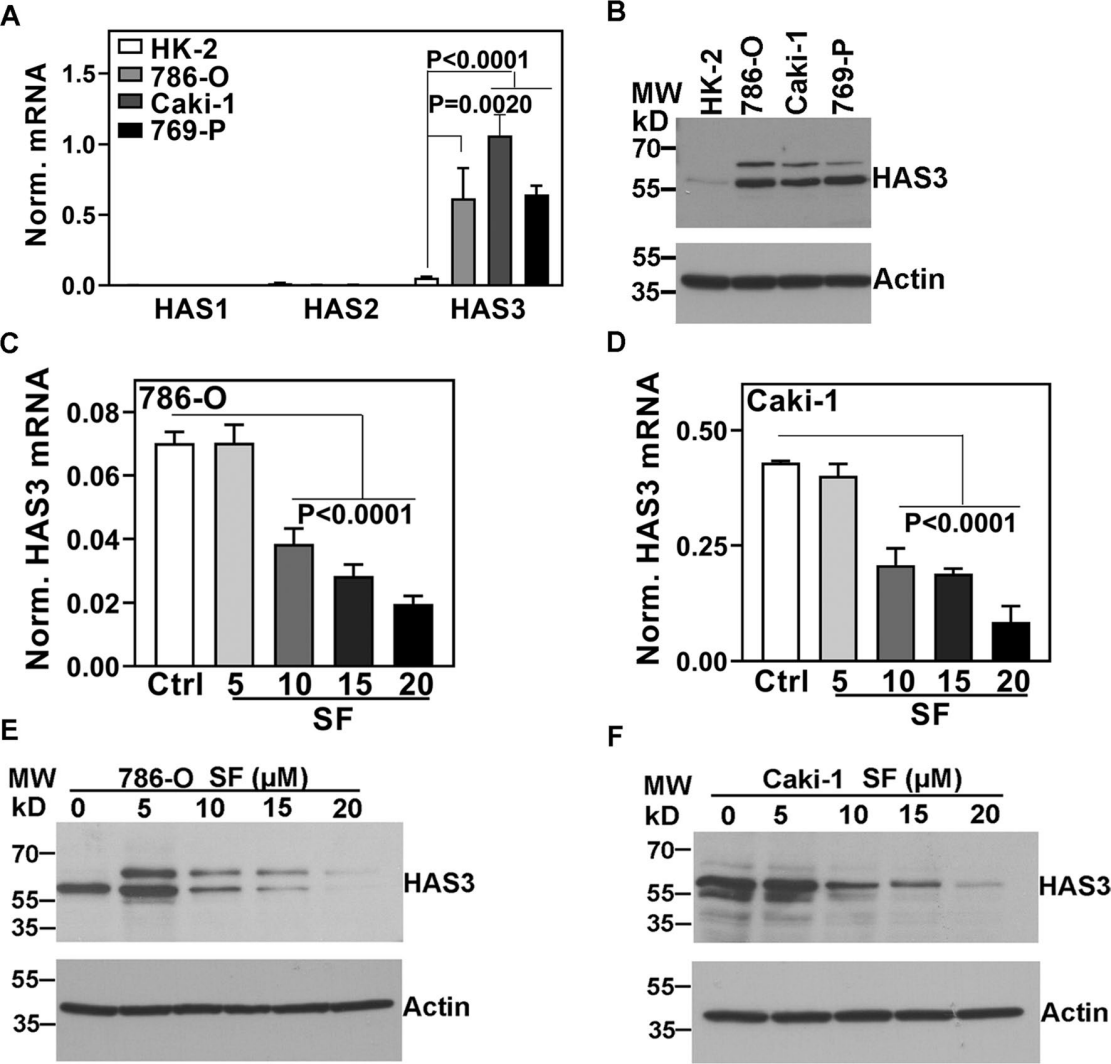
HA-synthase transcript levels and the effect of SF on HAS3 expression in RCC cells. **A**, **B**: HA-synthase transcript (A) and HAS3 protein (B) expression in HK-2 and RCC cells (786-O, Caki-1, 769-P). **C**–**F**: RCC cells treated with SF for 48 h were analyzed for HAS3 transcript levels (**C**, **D**) or HAS3 protein expression (**E**, **F**) by RT-qPCR and immunoblotting, respectively. Panels A, C and D: HAS3 transcript levels were normalized to β-actin. The images are cropped for brevity. Uncropped images of the blots shown in panels B, E, and F are provided in the Additional file 1 (Appendix). Data: Mean ± SD (n = 3 or 4). Panel B, E, F: actin as the loading control

To test the hypothesis that SF inhibits HA synthesis, we initially investigated the expression of all three HA-synthases in RCC cells. The expression of HAS1 and HAS2 was minimal in the normal kidney (HK-2) and RCC cell lines (Fig. 1A). However, HAS3 transcript and protein levels were > 20-fold elevated in both VHL + (i.e., Caki-1) and VHL- (786-O, 769-P) RCC cell lines, compared to HK-2 cells (Fig. 1A, B; Additional file 1: Table S3).

Von Hippel-Lindau (VHL) encodes for the substrate recognition component of an E3 ubiquitin ligase, and the well-characterized substrate of VHL is the hypoxia inducible factor (HIF) α subunit. HIF-α (HIF-1α or HIF-2α) is prolyl hydroxylated in normoxia, generating a high-affinity binding site for VHL. Once VHL binds HIF-α, it is ubiquitinated and rapidly degraded. Under hypoxia or when VHL is mutated, HIF-α is stabilized and binds to HIF-1β. When the HIF heterodimer translocates to the nucleus, it binds to the cis-acting hypoxia elements in the hypoxia-responsive genes and activates their transcription. Mutations and promoter hypermethylation in the VHL gene are associated with clear cell RCC [42, 43].

At concentrations ≥ 10 µM, SF alone downregulated HAS3 transcript and protein levels in a dose-dependent manner; at 20 µM dose, the decrease in HAS3 expression was > 80% (Fig. 1C–F; Additional file 1: Table S3). However, such high concentrations of SF are not pharmacologically achievable.

It is unclear why in immunoblots, the anti-HAS3 antibody detects a single protein band or a doublet and, in some cases, a triplet (Figs. 1E, F). We believe these bands are closely related to the HAS3 protein based on our data described below. Nevertheless, HA synthases are multi-pass transmembrane proteins in a lipid complex [44]. Therefore, the multiple HAS3 protein bands could be complexes, biosynthetic precursors, mature protein, and degradation products.

### HAS3 levels in RCC specimens correlate with metastasis and survival

**Fig. 2:**
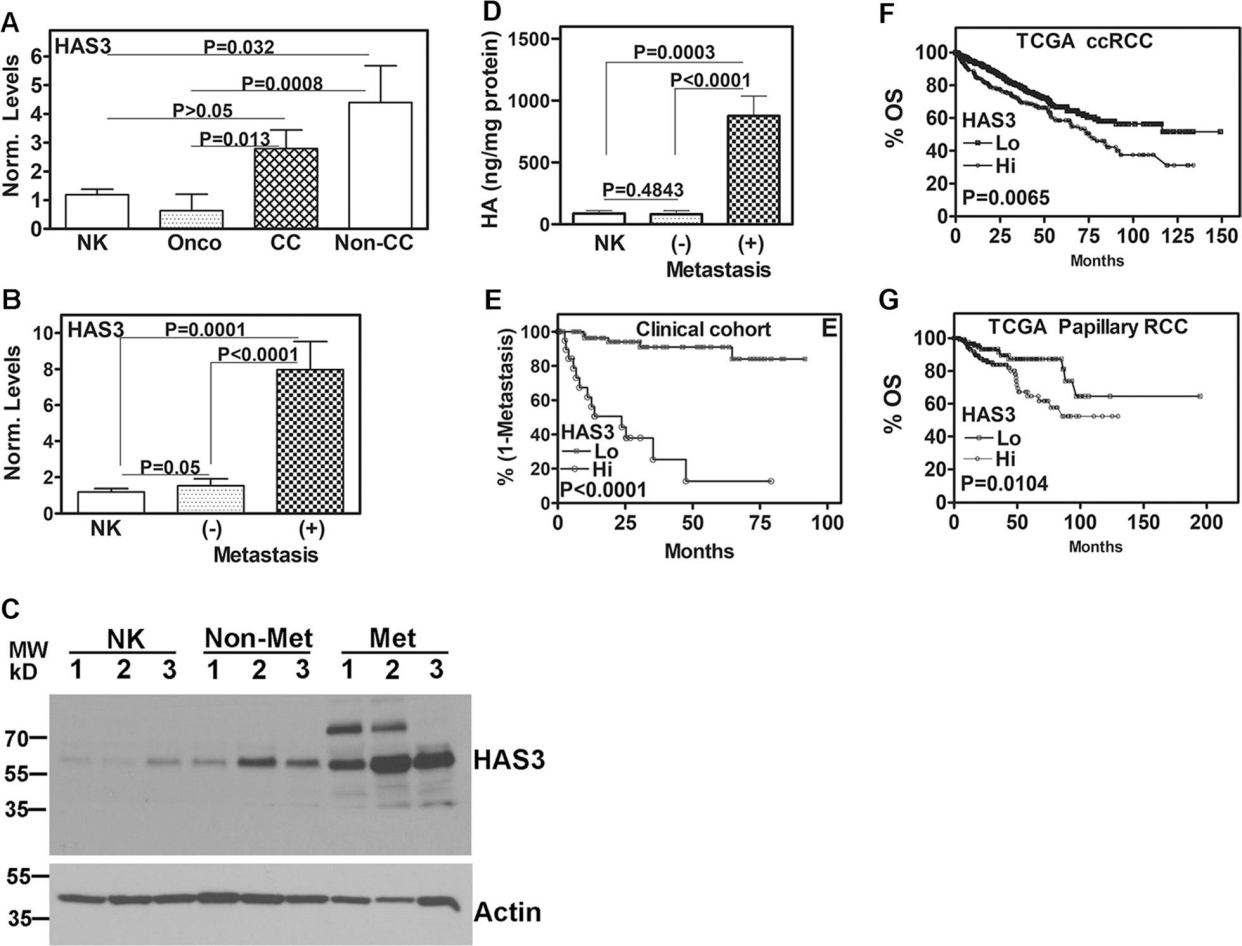
Measurement of HAS3 levels in normal and RCC tissues and their association with the clinical outcome. **A**, **B:** HAS3 transcript levels were measured in normal kidney (NK) and RCC tissues and normalized to TBP transcript levels. A: Data were stratified for NK, oncocytoma, clear cell (cc) and non-cc RCC (papillary, chromophobe, collecting duct, sarcomatoid) tissues. B: transcript data for RCC tissues were stratified based on the development of metastasis during follow-up. **C**, **D:** HAS3 protein expression (C) and HA levels in NK and RCC tissues based on the patients’ metastasis status during follow-up. Loading control: Actin. The images are cropped for brevity. Uncropped images of the blots shown in panel C are provided in the Additional file 1 (Appendix). Data: Mean ± SD (D). **E**–**G**: Stratification of the cohorts for metastasis (clinical; panel E) and OS (TCGA-KIRC, TCGA-KIRP; panels F, G) based on HAS3 mRNA levels. P values based on the log-rank test

Since SF is approved for the treatment of mRCC, we investigated if the upregulation of HAS3 in tumors that progress to metastasis is a reason why SF fails in the clinic. Specifically, do HAS3 levels in primary tumors predict the patient outcome regarding metastasis and survival? In a clinical cohort of 129 tissues (Additional file 1: Table S2), HAS3 transcript levels were elevated in ccRCC and non-ccRCC specimens compared to NK and oncocytoma specimens (Fig. 2A). The non-ccRCC pathologies were papillary, chromophobe, collecting duct, and a sarcomatoid variant. In tumor specimens of patients who had or developed mRCC, HAS3 transcript levels were about 8–tenfold elevated compared to those who did not develop mRCC during follow-up (Fig. 2B). The increase in HAS3 protein levels in metastatic tumors was > 20-fold compared to NK tissues and about fourfold higher than in non-metastatic tumors (Fig. 2C; Additional file 1: Table S3). HA levels were also > fivefold elevated in metastatic tissues compared to NK and non-metastatic tissues (Fig. 2D). HAS3 levels significantly predicted metastasis in the univariate analysis (P = 0.0004; OR: 1.29; 1.12–1.48). In multivariate analysis, HAS3 levels were an independent predictor of metastasis along with T-stage (Additional file 1: Table S4). High HAS3 levels significantly stratified patients to a higher risk for metastasis (Fig. 2E). HAS3 levels correlated with OS in the TCGA-KIRC (P = 0.0293; OR: 1.24; 1.02–1.52) and in the TCGA-KIRP (P = 0.015; OR: 1.31; 1.− 5–1.64) datasets. Kaplan–Meier plots showed that HAS3 levels also significantly stratified patients regarding the risk for death in the TCGA-KIRC and TCGA-KIRP cohorts (Fig. 2F, G).

### SF combination with MU downregulates HAS3 expression and HA synthesis in RCC cells

**Fig. 3:**
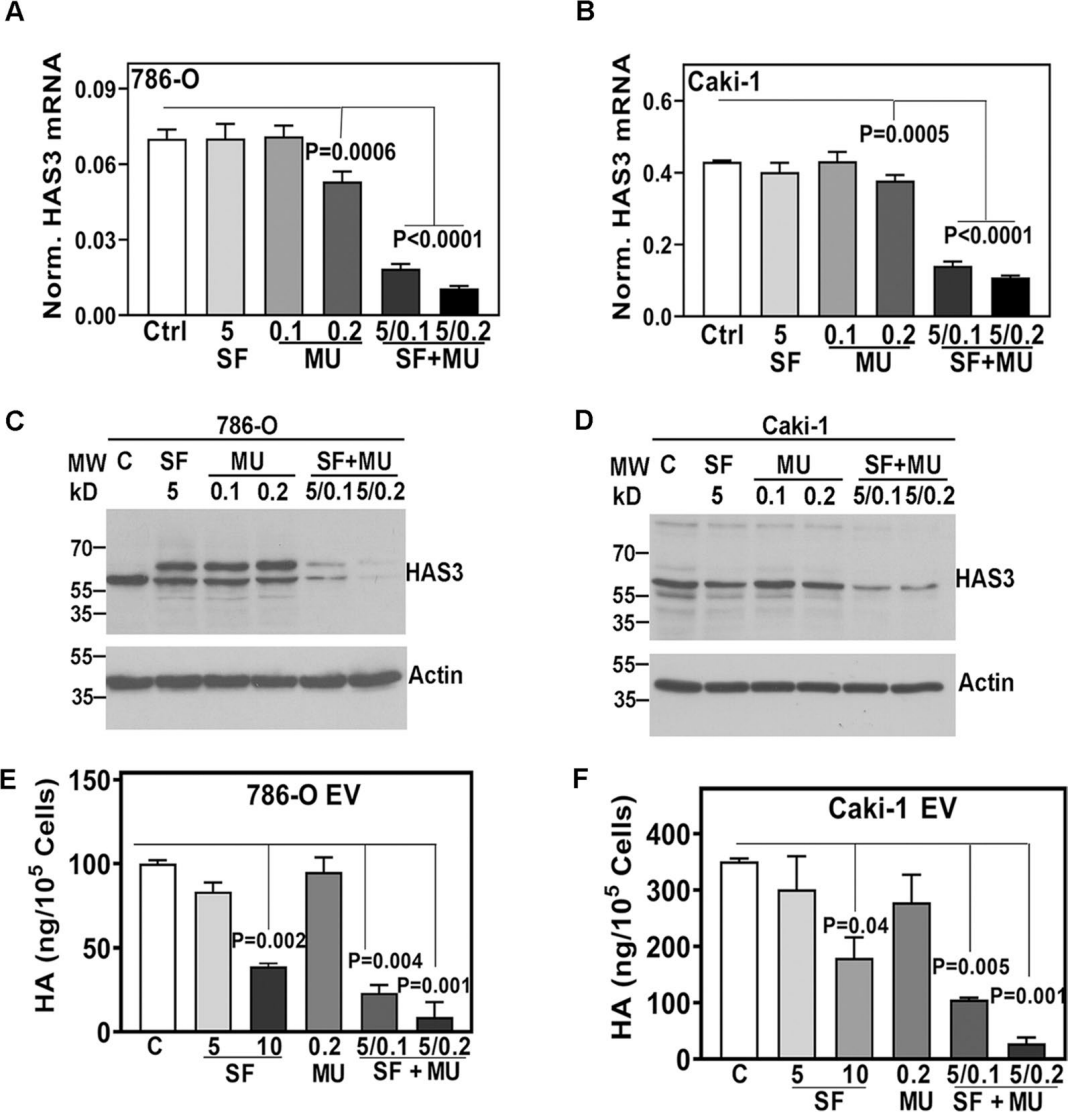
Effect of SF plus MU combination on HAS3 expression and HA levels in RCC cells. **A**–**F**: RCC cells were treated with SF, MU or their combination for 48 h and analyzed for HAS3 transcript levels (**A**, **B**), and protein expression (C, D). HAS3 transcript levels were normalized to β-actin. Data: Mean ± SD (n = 3). **C**, **D**: actin as the loading control. The images are cropped for brevity. Uncropped images of the blots shown in panels C and D are provided in the Additional file 1 (Appendix). Cell CM were assayed for HA levels by HA test and normalized to cell number (E, F). Note: Data shown are for the EV transfectants of 786-O and Caki-1. HA levels in the HAS3 transfectants are shown in Fig. 4

We previously showed that MU blocks SF inactivation by downregulating UGT1-A9 [21]. Since SF alone could downregulate HAS3 at ≥ 10 µM dose, we tested if, in combination with MU, SF could downregulate HAS3 at the pharmacological dose (5 µM). SF at the pharmacological dose and MU at low doses (0.1 and 0.2 mM) could not downregulate HAS3 transcript or protein levels. However, their combination (5/0.1; 5/0.2) downregulated HAS3 expression by > 90% (Fig. 3A–D; Additional file 1: Table S3). Similarly, the combinations (5/0.1, 5/0.2) inhibited HA synthesis > 90% in these cell lines (Fig. 3E, F). These results show that HA synthesis mediated by HAS3 is a possible target of the SF + MU combination.

### HAS3 is the target for the cytotoxic effects of SF alone (at higher doses), and SF and MU combination

**Fig. 4:**
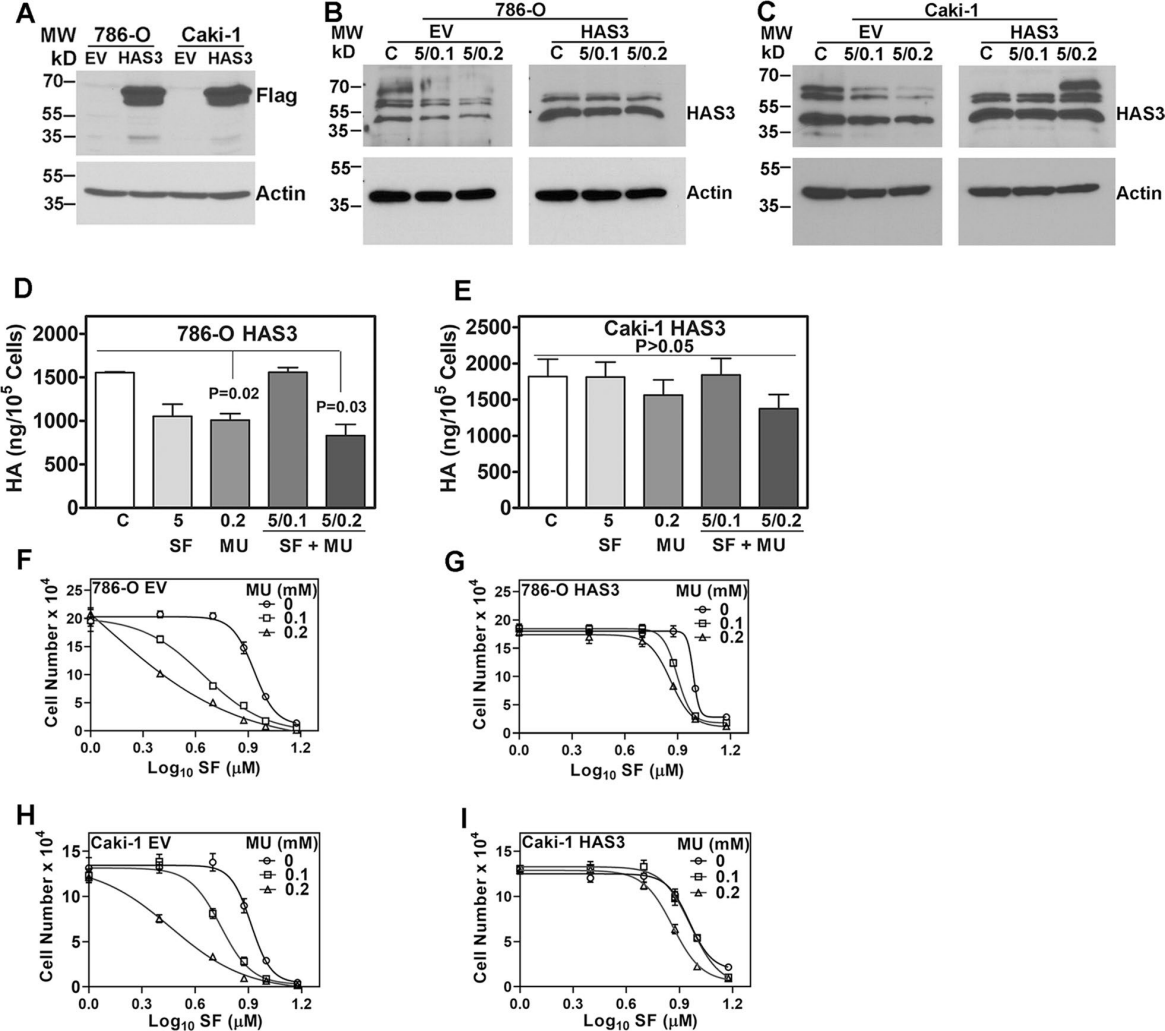
Ectopic HAS3 expression attenuates the effect of SF and MU combination on RCC cell growth. **A:** Expression of Flag-tagged HAS3 protein in RCC cells. **B**, **C:** HAS3 protein expression in RCC cell transfectants following treatment with SF and MU combination for 48–60 h. The images are cropped for brevity. Uncropped images of the blots shown in panels A–C are provided in the Additional file 1 (Appendix). **D**, **E**: Measurement of HA levels in HAS3 transfectants’ CM using the HA test. **F**–**I:** As indicated, RCC cells were treated with SF, MU, or the combination. Viable cells were counted at 72 h. Dose-responsive curves generated by variable slope equation and actual data points are shown. Data in panels **F**–**I**: Mean ± SD (n = 6 to 8)

To examine if HAS3 is the target of SF and SF + MU in RCC cells, we expressed HAS3 in 786-O and Caki-1 cells under a viral promoter. In these transfectants, neither SF nor the combination should downregulate HAS3 because the viral promoter is driving HAS3 expression. Due to their different VHL status and basal HA synthesis levels, we chose 786-O and Caki-1 cells for these studies. HAS3 transfectants expressed a Flag-epitope tagged HAS3 protein in both cell lines (Fig. 4A). As expected, the SF + MU combination downregulated HAS3 protein levels in EV transfectants but not in the corresponding HAS3 transfectants of RCC cells (Fig. 4B, C; Additional file 1: Table S3). Consequently, SF + MU caused only a modest reduction in HA levels secreted in the CM of HAS3 transfectants (Fig. 4D, E).

We examined the sensitivity of EV and HAS3 transfectants to SF in combination with MU. SF alone inhibited the growth of both 786-O and Caki-1 cells in a dose-dependent manner with an IC_50_ of > 8 µM in both cell lines (Fig. 4F, H; Table 1). MU lowered the IC_50_ of SF in both cell lines, less than the pharmacological dose (about 5 µM). The IC_50_ of SF for 786-O cells was 4.4 µM and 1.2 µM in the presence of 0.1- and 0.2-mM MU, respectively (Fig. 4F, Table 1). In Caki-1 cells, which produce higher HA amounts, the IC_50_ of SF was 2.9 µM in the presence of 0.2 mM MU (Fig. 4H, Table 1).

**Table 1:** IC_50_ values for the cytotoxicity of SF or SF plus combination

Transfectant	SF IC_50_ for EV/HAS3 transfectants		
	0 mM MU	0.1 mM MU	0.2 mM MU
786-O EV	8.586	4.405	1.201
786-O HAS3	9.736	7.897	7.254
Caki-1 EV	8.216	5.544	2.912
Caki-1 HAS3	9.043	9.194	7.266

SF IC_50_ for shRNA transfectants			
	Ctrl	HAS3 # 1	HAS3 # 2
786-O	8.386	1.974	3.049
Caki-1	7.385	3.120	2.792

IC_50_ values were calculated from the non-linear regression analysis of dose–response curves using the sigmoidal dose–response (variable slope) equation

We have shown that by blocking SF inactivation by UGT1-A9 downregulation, MU allows SF to exert its cytotoxic effects on RCC cells [21]. If the cytotoxic effects of SF are because it downregulates HAS3 and consequently inhibits the tumor-promoting effects of HA, then the ectopic expression of HAS3 under a viral promoter in RCC cells should make them resistant to the SF + MU combination. Indeed, in HAS3 transfectants, the IC_50_ of SF remained higher than the pharmacologically achievable dose (7.3–9.1 µM; Fig. 4G, I, Table 1).

**Fig. 5:**
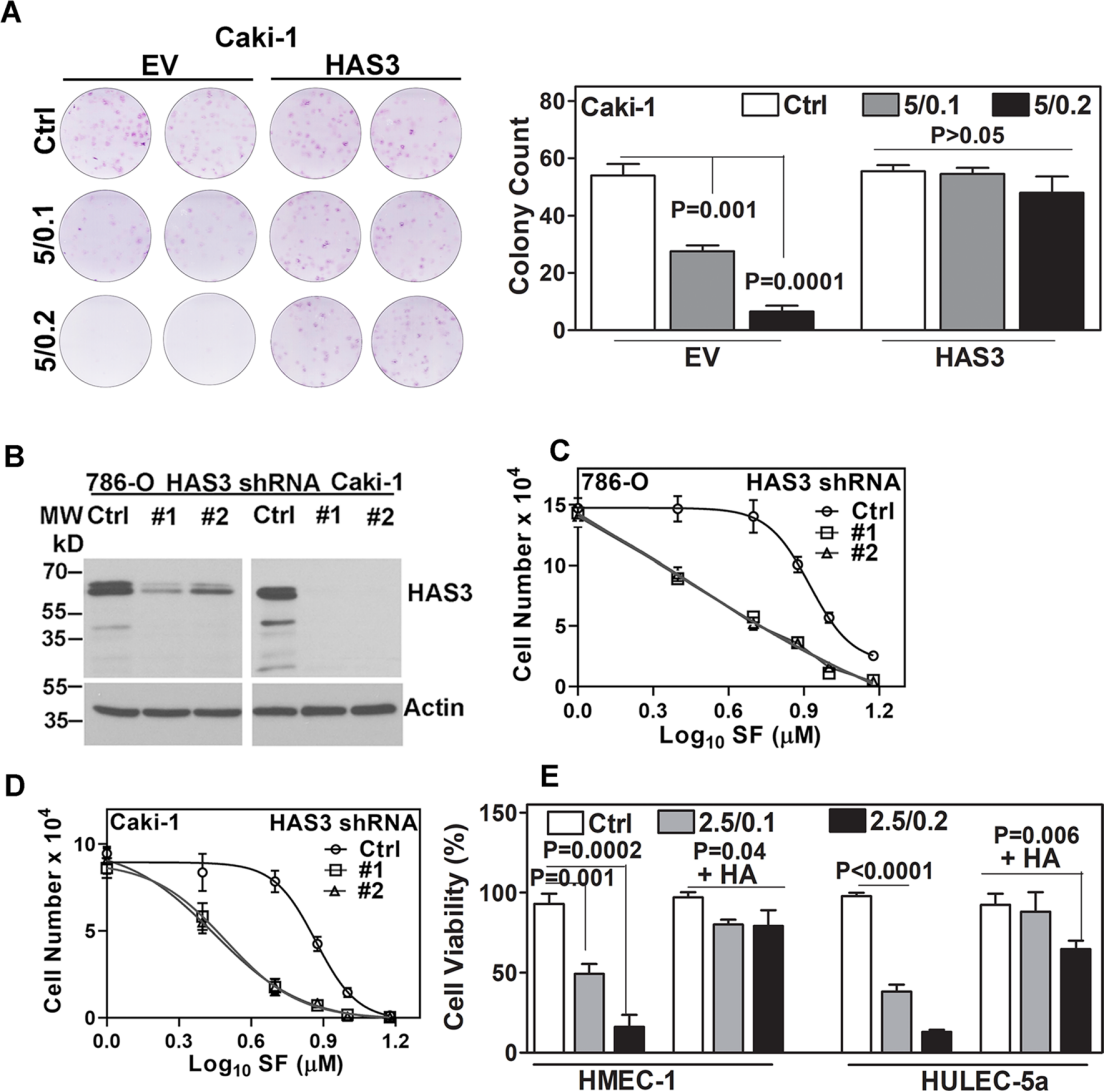
Clonogenic survival and proliferation of HAS3 and HAS3 knockdown transfectants treated with the combination. **A**: Clonogenic survival of Caki-1 EV and HAS3 transfectants following treatment (as indicated). Crystal violet staining (left panel) and quantification (right panel) of the colonies stained on day 7. **B**: HAS3 protein expression in HAS3 shRNA transfectants of RCC cells. Actin is the loading control. The images are cropped for brevity. Uncropped images of the blots shown in panel B are provided in the Additional file 1 (Appendix) **C**, **D**: Control and HAS3 shRNA transfectants of RCC cells were treated with SF and viable cells were counted after 72 h. **5E:** Viability of endothelial cells (HMEC-1, HULEC-5a) exposed to SF + MU in the presence or absence of HA was measured by MTT assay was performed at 72 h. Percent viability was calculated from untreated control (100%). Data in panels A and C–E: Mean ± SD (n = 3 to 8)

In a clonogenic survival assay, the combination of SF plus MU inhibited the clonogenic survival of EV cells by ~ 90%. However, it caused ≤ 10% inhibition of the clonogenic growth in the HAS3 transfectants (Fig. 5A). To confirm that the cytotoxic effects of SF in RCC cells are through HAS3 downregulation, we knocked down HAS3 expression in RCC cells by shRNA transfection (Fig. 5B). In the shRNA transfectants, the IC_50_ for growth inhibition for SF alone was 1.9–3.0 µM (Fig. 5C, D; Table 1). It is noteworthy the multiple bands present in the control shRNA transfectants were knocked down by HAS3 shRNA (Fig. 5B), demonstrating that these multiple bands detected by the anti-HAS3 antibody are different species of the HAS3 protein.

Tyrosine kinase inhibitors such as SF have potent antiangiogenic activity because endothelial cells express the known targets of SF (i.e., VEGF-receptor, PDGF-receptor B, c-kit, and c-Raf [12–14]). We have shown the presence of small angiogenic HA fragments in the tumor microenvironment [41]. Moreover, tumor-associated HA forms a protective coat that prevents drug penetration [25]. We hypothesized that HA produced by RCC cells (due to HAS3 expression) protects endothelial cells from SF's cytotoxic effects. Therefore, we tested if the presence of HA would affect the growth-inhibitory effects of SF + MU on human microvessel endothelial cells. We observed 85–90% inhibition of HMEC-1 and HULEC-5a growth by SF in the presence of MU. However, inclusion of HA during treatment attenuated the growth-inhibitory effects of SF + MU on endothelial cell growth (Fig. 5E). In the presence of HA, SF + MU (2.5/0.2) caused only 25–35% growth inhibition in endothelial cells.

### HAS3 expression attenuates the molecular markers of cell-cycle arrest and apoptosis in SF + MU-treated RCC cells

**Fig. 6:**
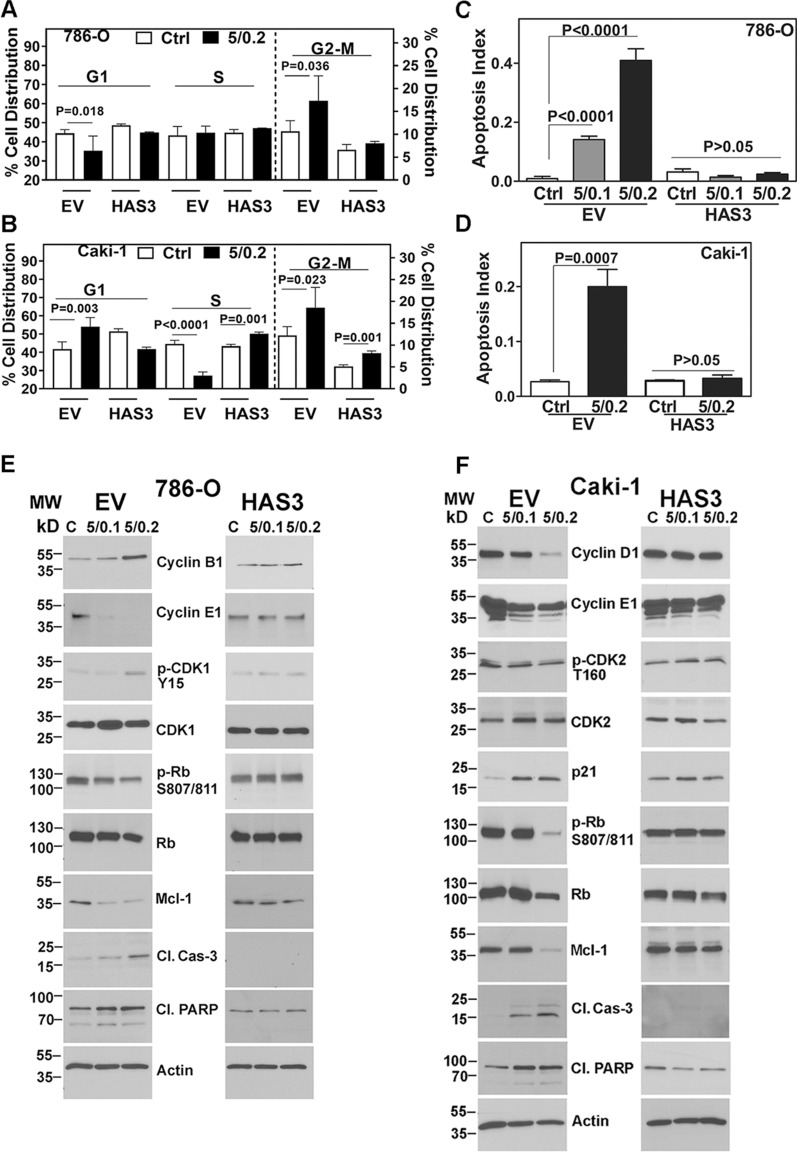
Cell-cycle and apoptosis analyses of RCC cell transfectants treated with the combination. **A**–**F:** Transfectants (EV, HAS3) were treated with SF and MU combination, as indicated. Treated cells were analyzed for cell cycle progression and cell-cycle phase markers (A, B, E, and F) and for apoptosis and related markers (**C**–**F**). Data in A–D: Mean ± SD (n = 3). Please note that for brevity and clarity p values are shown for those pairs where the difference is statistically significant. In panels E and F actin is the loading control. The images are cropped for brevity. Uncropped images of the blots shown in panels E and F are provided in the Additional file 1 (Appendix)

Consistent with the growth inhibition, the SF and MU combination induced cell cycle arrest in EV transfectants. While G2-M arrest was observed in the EV transfectants of 786-O cells, Caki-1 EV cells were arrested in both the G0-G1 and G2-M phases following the treatment (Fig. 6A, B). As expected, SF + MU treatment caused minimal cell cycle arrest in HAS3 transfectants of both RCC cells (Fig. 6A, B). Analyses of cell-cycle markers revealed a > threefold increase in cyclin-B1 and phospho (p)-cdk1 levels and a 2–tenfold decrease in cyclin-E1 and p-Rb levels following the treatment of 786-O EV transfectants with SF + MU (Fig. 6E; Additional file 1: Table S3). In Caki-1 EV cells, the treatment caused a 2–tenfold reduction in cyclin-D1 cyclin-E1, p-cdk2, and p-Rb levels and a 7.6-fold upregulation of p21 levels, confirming cell-cycle arrest in G0-G1 and G2-M phases (Fig. 6F; Additional file 1: Table S3).

The treatment of 786-O and Caki-1 cells with SF and MU combination induced cell-cycle arrest and a > tenfold increase in apoptosis. However, the treatment failed to induce apoptosis in the corresponding HAS3 transfectants (Fig. 6C, D). The analysis of apoptosis markers confirmed these results. The combination caused a tenfold downregulation of the anti-apoptotic marker Mcl-1 and an upregulation of cleaved caspase-3 (activated caspase-3) and cleaved PARP in the EV transfectants. However, SF + MU treatment caused only a marginal alteration in these markers in the HAS3 transfectants (Fig. 6E, F; Additional file 1: Table S3).

### Ectopic HAS3 expression overcomes the inhibitory effects of SF + MU combination on RCC cell motility and invasion.

**Fig. 7:**
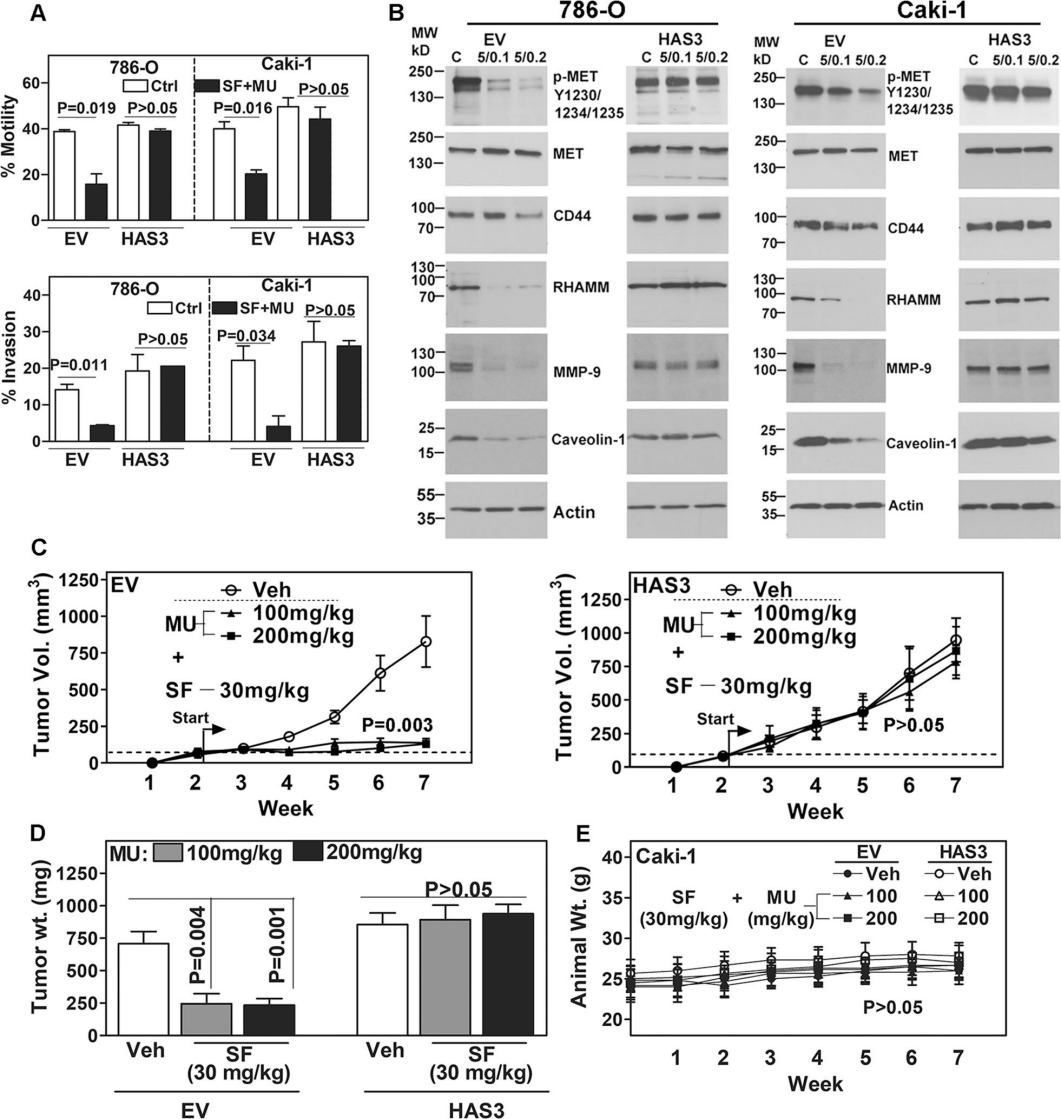
Analyses of anti-invasive and anti-tumor activities of SF and MU combination in EV and HAS3 transfectants. **A**: RCC cell transfectants treated with the combination were analyzed for chemotactic motility (at 18 h; top panel) and for invasive activity (at 48 h; bottom panel), respectively. Data: Mean ± SD (n = 3). **B**: The transfectants were also analyzed for markers associated with RCC invasion/metastasis; actin serves as the loading control (B). The images are cropped for brevity. Uncropped images of the blots shown in panel B are provided in the Additional file 1 (Appendix). **C**–**E**: In an athymic xenograft model, Caki-1 tumors (EV and HAS3) were established. Starting at two-weeks, mice were treated with vehicle or SF and MU combination, until week seven; six mice per group; 2 males and 4 females. Tumor volume and animal weight were measured weekly (**C**, **E**) and tumor weight was measured at endpoint (**D**). Data in panels C–E: Mean ± SEM

Increased levels of tumor-associated HA promote tumor invasion and metastasis by signaling through its receptors CD44 and RHAMM [45–47]. Our published study showed that CD44 and RHAMM are potential predictors of mRCC with high efficacy [40]. Consistent with the inhibition of HA synthesis, the SF and MU combination decreased chemotactic motility by 50–60% and invasive activity by 80% in EV transfectants of both 786-O and Caki-1 cells (Fig. 7A). However, in the HAS3 transfectants with ectopic HAS3 expression, the SF + MU combination caused < 10% inhibition of the chemotactic motility and invasion (Fig. 7A).

To confirm that the combination inhibits chemotactic motility and invasion through SF-mediated downregulation of HAS3 and the inhibition of HA synthesis, we analyzed the molecules associated with HA-signaling [27, 45, 48, 49]. Inhibition of HA synthesis is known to downregulate its receptors CD44 and RHAMM and an associated invasive signature consisting of c-MET activation (phosphorylation at tyrosine 1230/1234/1235) and matrix metalloproteinase-9 (MMP-9) and caveolin-1 expression [21, 27, 35]. Consistent with the inhibition of HA synthesis, SF and MU combination caused a 2 to 22-fold reduction in the levels of HA receptors and associated signaling molecules in EV cells. However, in the HAS3 transfectants, the combination failed to block HA-signaling (Fig. 7B; Additional file 1: Table S3).

### SF + MU combination is ineffective in inhibiting the growth of ectopic HAS3-expressing Caki-1 tumors

**Fig. 8:**
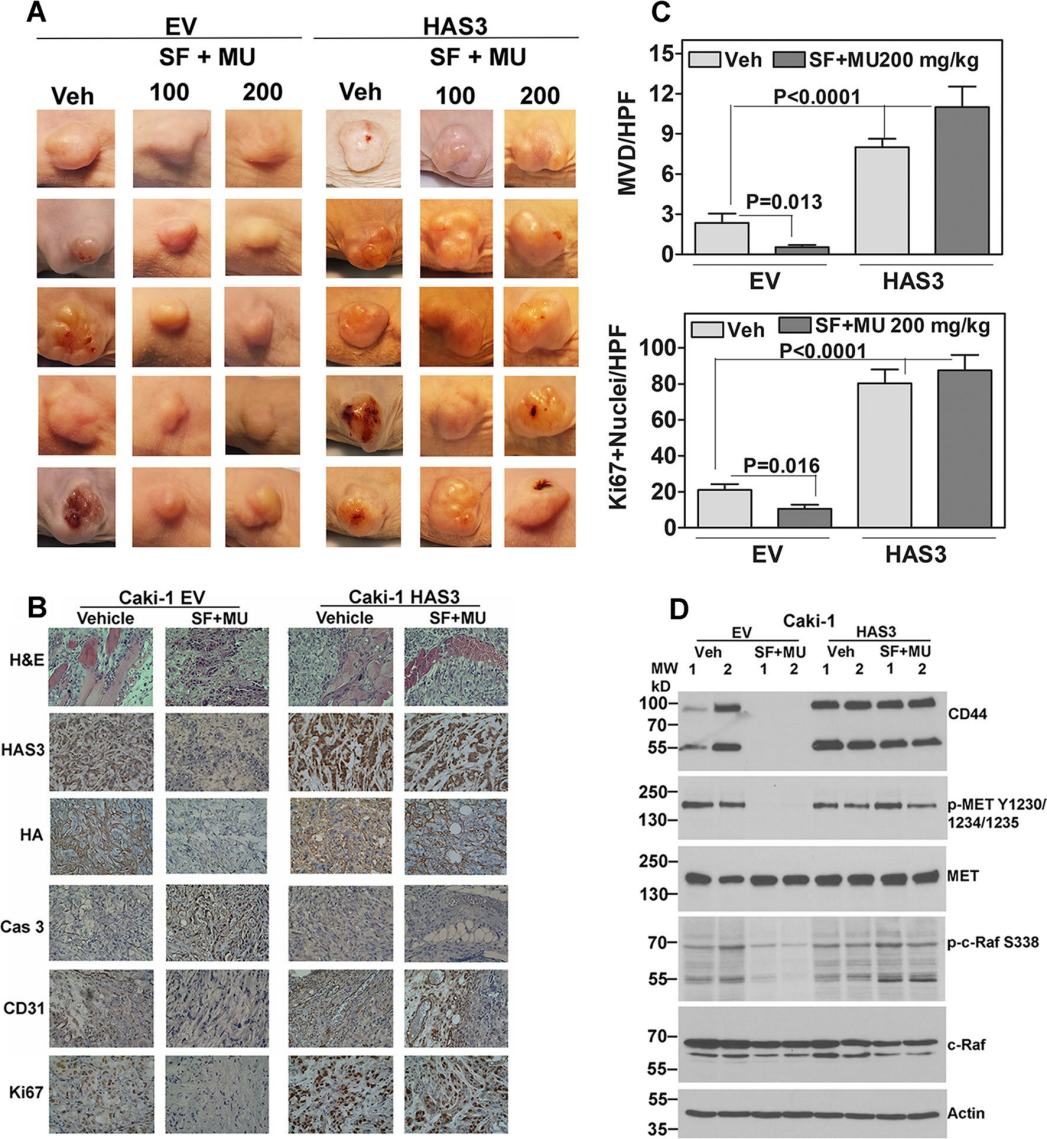
Analysis Caki-1 subcutaneous tumors. **A**: Caki-1 subcutaneous tumor histology images. Tumor photos were taken at necropsy. For these photos, tumor volume and weight data are shown in 7C and D. Three photos per group are shown. Caki-1 tumors in the vehicle (both EV and HAS3 tumors) and combination (HAS3 tumors only) show invasion into muscle and the fat layers. IHC shows HAS3, HA and Ki67 (proliferation) expression, microvessels (CD31 staining), and apoptosis indicator, cleaved caspase 3 staining; magnification: 400X. **B**, **C**: Microvessels and Ki67 positive nuclei were counted at 400X magnification to determine microvessel density (MVD) and proliferation index (Ki67 positive nuclei/high power field (HPF)), respectively. Data: Mean ± SD; n = 10. **D**: Extracts of two randomly chosen Caki-1 subcutaneous tumors from each treatment group were analyzed for indicated proteins; actin is the loading control. The images are cropped for brevity. Uncropped images of the blots shown in panel D are provided in the Additional file 1 (Appendix)

We have previously demonstrated the effect of SF and MU combination in the Caki-1 subcutaneous and orthotopic models. Likely because of higher HA levels in the Caki-1 cells, these tumors do not respond to SF treatment [35, 50]. Therefore, this is a suitable model to test combinations that could potentially overcome unresponsiveness to SF. Consistent with MU increasing SF’s efficacy by blocking its inactivation, the combination inhibited Caki-1 tumor growth (Figs. 7C, D;  8A). In comparison to the EV tumors, where the combination inhibited tumor growth by > 70%, ectopic-HAS3-expressing Caki-1 tumors (established by implanting HAS3 transfectants) were resistant to the treatment (Figs. 7C, D; 8A). The combination did not affect animal weight (Fig. 7E). We have shown that the combination has minimal toxicity [21, 35]).

Histology confirmed the invasive and angiogenic nature of the Caki-1 tumors (Fig. 8A, B; EV: vehicle group; HAS3: vehicle and treatment groups). While EV tumors in the combination treatment group demonstrated increased apoptosis, as indicated by elevated cleaved caspase-3 staining, these tumors showed decreased proliferation index (Ki67 positive nuclei), microvessels and HAS3 and HA expression. HAS3 tumors showed a 3–fourfold higher MVD, proliferative index, and high HAS3 and HA expression compared to EV tumors; however, the treatment failed to inhibit angiogenesis or to reduce HAS3/HA expression in these tumors (Fig. 8B, C). The analyses of molecules associated with HA signaling further confirmed that HAS3-mediated HA synthesis is the target of SF in RCC. Specifically, as shown in Fig. 8D, EV tumors from the SF + MU combination treatment group showed decreased levels of HA receptors, MET-activation, and c-Raf-activation (phosphorylation of Raf at Serine-338); c-Raf is the target of SF in endothelial cells [50]. The treatment did not cause a reduction in the levels of these signaling molecules in HAS3 tumors (Fig. 8D, Additional file 1: Table S3).

### Ectopic HAS3 expression abolishes the ability of SF + MU combination to inhibit mRCC

**Fig. 9:**
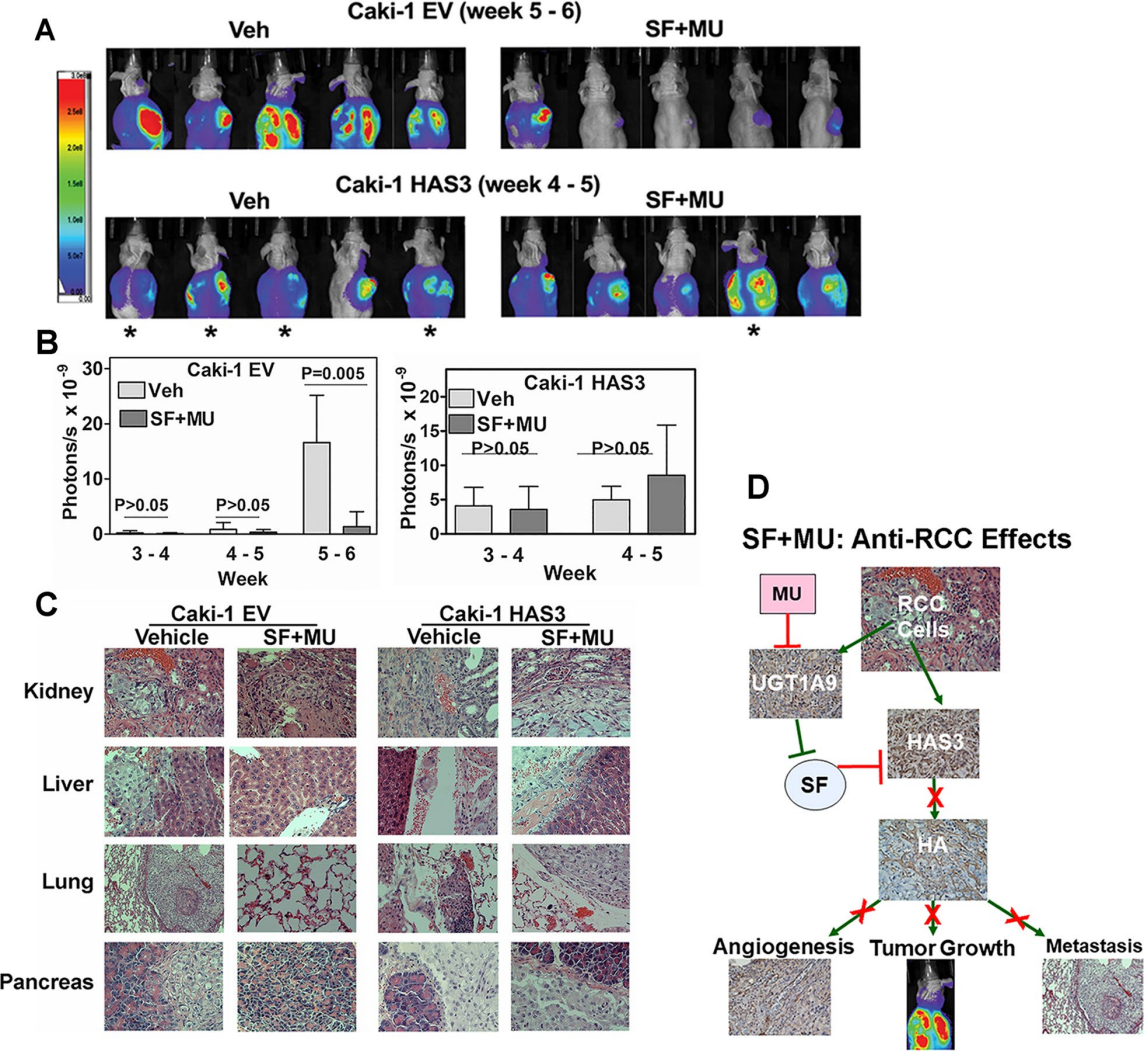
Effect of the combination on orthotopic kidney tumor growth and metastasis and a schematic model of the mechanism of SF and MU combination for mRCC. **A**, **B**: Orthotopic kidney tumors were established in athymic mice (n = 5; males) from Caki-1 (EV, HAS3) transfectants. From day nine, animals were treated with vehicle or SF (30 mg/kg) and MU (200 mg/kg) combination, until visible metastasis in the vehicle group of EV cells and in HAS3 groups (both vehicle and treatment). Tumor development as monitored by bioluminescence imaging. **C**: Tissue histology to evaluate kidney tumors, metastasis, and organ toxicity (treatment group). In mRCC, **D**: Schematic models: Tumor cells express high levels of both HAS3 and UGT-1A9. Elevated HAS3 expression drives RCC growth and metastasis. By downregulating HAS3 expression, SF can potentially halt mRCC. However, elevated UGT-1A9 levels inactivate SF, and therefore, at pharmacological dose, SF is ineffective against mRCC. Low dose MU downregulates UGT-1A9 and prevents the inactivation of SF by RCC cells. Therefore, SF + MU combination shows high efficacy in inhibiting/eliminating RCC growth and metastasis in preclinical models

We have shown that luciferase-expressing Caki-1 (Caki-1-luc) is a reliable spontaneously metastatic orthotopic mouse model of RCC [21]. In agreement, in the vehicle treatment group implanted with the Caki-1 EV-luc transfectant, large kidney tumors were visible on bioluminescence imaging within 5–6-weeks (Fig. 9A, B). In the EV-luc group treated with the combination, tumor growth was not detected in 60% of the mice; in the remaining mice, tumor growth was reduced > tenfold (P = 0.005; Fig. 9B); excluding one outlier (by Grubb's test), the tumor growth in the remaining mice was > 100-fold lower. While all mice in the vehicle group developed visible metastasis, only one mouse in the treatment group developed metastasis, i.e., an 80% reduction in the development of mRCC (Fig. 9A). Consistent with the role of HA in promoting tumor growth and metastasis [49], mice implanted with HAS3-luc transfectant developed visible kidney tumors within three weeks and mRCC in < 5 weeks (Fig. 9A, B). Evidence of mRCC in various organs, such as the liver and the gastrointestinal tract (liver, pancreas), was confirmed by histology in the vehicle groups of mice implanted with EV-luc or HAS3-luc cells (Fig. 9C). However, while the treatment abrogated mRCC in mice implanted with EV-luc cells, it failed to do so in HAS3-luc tumor-bearing mice (Fig. 9C).

## Discussion

Our recent study demonstrated that by downregulating UGT1-A9, MU re-sensitizes preclinical models of RCC to SF. That study provided a proof-of-concept that a less effective drug could regain efficacy when combined with a minimally toxic agent that targets the primary mechanism of resistance to the drug [21]. The discovery of HAS3 as a new SF target explains why SF is cytotoxic to RCC cells despite the apparent lack of expression of known SF targets in RCC cells [12–14]. It is well documented that in cell culture studies, SF alone, at a concentration higher than the pharmacological dose, shows antitumor activities [17–19]. Our discovery that SF downregulates HAS3 expression and HA synthesis shows that SF is both an anti-RCC and an antiangiogenic drug. SF effectively blocks HA synthesis by downregulating HAS3 at doses higher than pharmacologically achievable. A higher dose of SF is required because RCC cells (especially with high metastatic potential) express UGT1-A9, which inactivates SF by adding a glucuronic acid residue (i.e., glucuronidation). MU downregulates UGT1-A9 and enhances the activity of SF (Fig. 9D).

It is well-established that tumor-associated HA promotes tumor growth, especially tumor metastasis, aids tumor cells in avoiding immune surveillance, and protects them from cytotoxic agents by forming a protective coat [36, 45]. Among the three HA-synthases, HAS3 is the most catalytically active enzyme [51]. Our data show that in RCC cells and tissues, HAS3 is elevated and exists in multiple forms. It remains to be determined if these multiple forms represent posttranslational modifications or degradation products. Tumor-associated HA promotes epithelial-mesenchymal transition by signaling through its receptors (CD44, RHAMM) [27, 47, 49, 52–54]. In a feedback mechanism, a combination of SF with MU decreases HA synthesis and downregulates CD44 and RHAMM expression [21]. Increased HA synthesis in HAS3 transfectants overcomes the downregulation of CD44 and RHAMM by the SF + MU combination, demonstrating a driver role of HA in this feedback mechanism. Since HAS3 expression is under an ectopic (viral) promoter in these transfectants, it confirms that HA synthesis mediated by HAS3 is the primary target of the SF + MU combination in RCC. SF + MU also downregulates c-MET signaling and the downstream MMP-9 and caveolin-1 expression. Therefore, if SF remains active, it can be an effective drug, as it severely curtails the metastatic potential of RCC cells driven by tumor-associated HA signaling. The addition of MU achieves this objective.

The action of SF + MU on endothelial cells shows that the combination is effective as an antiangiogenic drug. Indeed, in tumor tissues, SF + MU inhibits the growth of microvessels. We have also demonstrated that the SF + MU combination inhibits microvessel endothelial cell growth and capillary formation [35]. Three signaling pathways in endothelial cells contribute to angiogenesis – VEGFR-Mek-Erk axis, c-Met signaling, and CD44-RHAMM-angiogenic HA fragment interaction. We have shown that SF + MU downregulates all three pathways in endothelial cells [35]. Our observation that adding HA protects endothelial cells against the combination is consistent with the known effects of tumor-associated HA on angiogenesis [36, 45]. These effects could be direct, where HA promotes CD44/RHAMM and CD44/c-Met signaling. Still, they could also be indirect (VEFGR-2 signaling), where HA forms a protective coat around endothelial cells which cannot be penetrated by drugs such as SF.

The Caki-1 model is resistant to SF [50], and our data show that it is an excellent model for studying mRCC. The SF + MU combination inhibits primary tumor growth and abrogates metastasis in this model. The resistance of the ectopically HAS3-expressing tumors to this combination further demonstrates that HAS3/HA is the primary target of the SF + MU combination. It is noteworthy that MU doses (100 and 200 mg/kg) are 569–1138 mg human equivalent dose at 70 kg, as mice have 12.3 times higher metabolic rate than humans [55]. In clinical trials, MU has been tested at doses higher than these [22]. Since SF is FDA-approved and MU is available as an over-the-counter drug, our study warrants clinical testing of this combination for mRCC treatment.

## Conclusions

Our present study highlights several significant aspects of personalized medicine for mRCC. Specifically, our study shows why a drug such as SF fails in the clinic but can be made effective through mechanism-based combinations. The impact of our study is the discovery of HAS3, a principal target of SF in RCC cells. Furthermore, HAS3 is highly expressed in RCC cells and potentially predicts poor clinical outcome in patients with RCC. Moreover, although SF could be highly effective against mRCC, its suboptimal efficacy is likely because of its inactivation by UGT1-A9, which is highly expressed in mRCC. The combination of SF with MU prevents its inactivation in RCC cells [21]. Therefore, the combination effectively inhibits RCC growth and metastasis. SF is already an approved mRCC drug. MU improves the efficacy of SF at 0.57 to 1.13 g per day human equivalent dose and has a favorable toxicity profile. Therefore, the translation of the SF and MU combination for the treatment of mRCC should be plausible.

The international guidelines currently recommend newer TKIs (e.g., Cabozantinib, Lenvatinib) rather than SF in the treatment of mRCC. Nevertheless, the median survival of patients with mRCC remains poor. Our study shows that mechanism-based combinations that overcome the resistance of RCC cells towards these newer TKIs may also improve their therapeutic efficacy against mRCC.

## In vivo studies

In vivo xenograft studies were approved by the Institutional Review Board of Augusta University (protocol # 2015–0738; date of approval: 07/21/2015; date of latest approval: 04/14/2022).

## Supplementary Information

**Additional file 1**: **Table S1.** Materials used in this study. Description of antibodies, reagents and primers used in this study. **Table S2. **Characteristics of clinical and TCGA RCC cohorts. Clinical cohort consists of 129 specimens acquired from 83 RCC patients (46 normal; 83 tumor). TCGA-KIRC and TCGA-KIRP datasets were downloaded from UCSC Xena (Xena functional explorer) Mean ± SD and median are reported. OS: (−) designates survival, (+) designates death. **Table S3.** Relative intensities of the immunoblot data presented in various figures. For each sample the normalized value (Intensity of the protein of interest ÷ intensity of loading control) was obtained. The normalized value in a treated sample (e.g., SF+MU doses: 5/0.1, 5/0.2) was divided by the corresponding normalized value in the control sample (or Veh) to obtain the fold change value. Therefore, for the control, the fold change was equal to 1. **Table S4.** Multivariate analysis to determine the relationship of clinical parameters and HAS3 levels to metastasis and OS. Cox Proportional Hazards Model was used to evaluate the ability of clinical parameters and HAS3 expression to associate with metastasis in the clinical cohort and with OS in TCGA cohort. Parameters included: Age, sex, tumor size, T-stage, low/high grade, lymphovascular invasion, renal vein invasion, HAS3. Renal vein invasion and tumor size data were not available in TCGA dataset, but M-stage was included. Only the parameters that reached significance are shown.

## Data Availability

Appropriate data are presented in the article and/or in Additional file 1; any additional information is available on request from the corresponding author.

## Abbreviations

ccRCC: Clear cell renal cell carcinoma
EV: Empty vector
HA: Hyaluronic acid
HAS3: Hyaluronic acid synthase 3
mRCC: Metastatic renal cell carcinoma
MU: 4-Methylumbelliferone
NK: Normal kidney
Onco: Oncocytoma
RCC: Renal cell carcinoma
SF: Sorafenib
TKI: Tyrosine kinase inhibitor
