# Supplementary material for: Targeting hyaluronic acid synthase-3 (HAS3) for the treatment of advanced renal cell carcinoma
Source: Cancer Cell Int. 2022 Dec 29;22:421. doi: 10.1186/s12935-022-02818-1 (PMC9801563; doi:10.1186/s12935-022-02818-1)

Targeting Hyaluronic Acid Synthase-3 (HAS3) For Treatment of Advanced Renal Cell Carcinoma

Wang JW and Jordan AR et al

Supplementary Materials

Supplementary Table 1: Materials used in this study. Description of antibodies, reagents and primers used in this study.

**Antibodies used in Western Blot analysis**						
**Target**	**Host species**	**Supplier**	**Catalog**	**Clone**	**Lot #**	**Dilution**
Actin	Goat	Santa Cruz Biotechnology	sc-1615 HRP	C11	J0914	1:20,000
Cleaved Caspase-3	Rabbit	Cell Signaling Technology	#9661	D175	43	1:1,000
Caveolin-1	Rabbit	Cell Signaling Technology	#3267S	D64G3	2	1:2,500
HCAM	Mouse	Santa Cruz Biotechnology	SC-7297 HRP	DF1485	F0816	1:500
CD44v6	Mouse	R&D Systems	BBA13	2F10	5781	1:1,000
Cdk1 Ab-3 (Cocktail)	Mouse	Neomarkers	MS-275-P1	A17.1.1+ POH-1	275P212C	1:1,000
Phospho-cdc2 (Tyr15)	Rabbit	Cell Signaling Technology	#4539	10A11	2	1:1,000
Cdk2 Ab-4	Mouse	Neomarkers	#MS-617-P1ABX	2B6 + 8D4)	617X310A	1:2,000
Phospho-CDK2 (Thr160)	Rabbit	Cell Signaling Technology	#2561	Polyclonal	2	1:1,000
Cyclin B1	Mouse	Santa Cruz Biotechnology	sc-245	GNS1	G0819	1:7,500
Cyclin D1	Rabbit	Abcam/Epitomics	1677-1(ab40754)	EP272Y	GR106612-1	1:2,000
Cyclin E1	Rabbit	Epitomics	3327-1(ab133266)	EPR194	YH052101C	1:2,000
FLAG	Mouse	Sigma-Aldrich	F1804	M2	SLBF6631	1:2,000
Custom Polyclonal anti-HAS3v1	Rabbit	GenScript	N/A	N/A	N/A	1;1,500
Mcl-1	Rabbit	Epitomics	#1239-1	D2W9E	1	1:1,000
Met (NT)	Rabbit	EMD Millipore	04-1051	EP1454Y	NG1944105	1:5,000
Phospho-Met (Tyr1230/Tyr1234/Tyr1235)	Rabbit	Millipore Sigma	07-810	Polyclonal	2766394	1:5,000
MMP-9	Rabbit	Epitomics	#2551-1	EP1254	YF-08-29-09C	1:3,000
p21 Waf1/Cip1	Mouse	Cell Signaling Technology	#2946	DCS60	5	1:2,000

Cleaved PARP (Asp214)	Rabbit	Cell Signaling Technology	#9541	D64E10	8	1:1,000
RAF1 (c-Raf)	Rabbit	ABclonal	A0223	Polyclonal	0014820201	1:2,000
Phospho-RAF1-S338	Rabbit	ABclonal	AP0498	Polyclonal	2101590101	1:1,000
Rb (4H1)	Mouse	Cell Signaling Technology	9309	4H1	9	1:2,000
Phospho-Rb (Ser807/811)	Rabbit	Cell Signaling Technology	9308	Polyclonal	12	1:1,000
RHAMM (CD168)	Mouse	Novocastra	NCL-CD168	2D6	6021453	1:300

Antibodies used in IHC

Target	Host species	Supplier	Catalog	Clone	Lot#	Dilution
Ki67	Rabbit	Abcam	Ab16667	SP6	GR3228859-3	1:240
CD31	Rabbit	Abcam	Ab28364	Polyclonal	6R272058-5	1:120
Custom Polyclonal anti-HAS3v1	Rabbit	GenScript	N/A	N/A	N/A	1:100

Other Reagents

Reagents and Kits	Supplier	Catalog #
RNeasy Mini Kit	QIAGEN	74104
iScript™ cDNA Synthesis Kit	BIO RAD	1708891
SsoFast™ Evagreen® Supermix	BIO RAD	1725204
RPMI 1640; DMEM; EGM-2	Thermo Fisher; Fisher Scientific; Millipore Sigma	MT10040CV; SH30243FS C-22011
Sorafenib, p-toluenesulfonate salt	LC Laboratories	SC-8502
ITS (Insulin, transferrin, selenium) solution	Sigma-Aldrich	I3146
4-Methylumbelliferone sodium salt	Sigma-Aldrich	M1508
Cell Death ELISA^{PLUS} Kit	CELLDETH-RO Roche; Sigma-Aldrich	11774425001
Crystal Violet	Sigma-Aldrich	C0775
Propidium Iodide	Sigma-Aldrich	P1470
Thiazolyl Blue Tetrazolium Bromide	Sigma-Aldrich	M5655
Cell Death Detection Elisa	Roche Diagnostics GmbH	11544675001
Corning™ Matrigel™ Matrix	Fischer Scientific	CB-40234
VivoGlo™ Luciferin	Promega Life Sciences	P1041

Transcript	Forward primer	Reverse primer
Actin	CAACTGGGACGACATGGA	GTTGGCCTTGGGGTTCAG
TBP	TGCACAGGAGCCAAGAGTGAA	CACATCACAGCTCCCCACCA
18S	AGGATGAGGTGGAACGTGTG	GGCTAGGACCTGGCTGTATTT
HAS3	CTCTACTCCCTCCTCTATATGT C	AACTGCCACCCAGATGGA
**shRNA**	**Supplier/Catalog**	**Sequences**
HAS3	OriGene Technologies/TR304150A (HAS3 shRNA#1)	GCTTATTGCCAGGACCTGTTCAGTG AGAC
	OriGene Technologies/TR304150B (HAS3 shRNA#2)	GCCTGCCAGTCCTACTTTGGCTGTG TGCA

Supplementary Table 2: Characteristics of clinical and TCGA RCC cohorts. Clinical cohort consists of 129 specimens acquired from 83 RCC patients (46 normal; 83 tumor). TCGA-KIRC and TCGA-KIRP datasets were downloaded from UCSC Xena (Xena functional explorer) Mean ± SD and median are reported. OS: (-) designates survival, (+) designates death.

Parameter	Clinical cohort	TCGA ccRCC cohort	TCGA papillary cohort
Number of specimens	Normal kidney (NK): 46<br>Tumor: 83 (RCC: 77; Oncocytoma: 6)	Tumor: 542	Tumor: 291
Age (years)	63.1 ± 14.1 years; median: 64 years.	60.6 ± 12.1 years.; median 61 years.	61.5 ± 12.1 years.; median 61 years.
Gender	Male: 59; Female: 23; Unknown: 1	Male: 351; Female: 191	Male: 214; Female: 77
Tumor type	Clear cell: 58; Non-clear cell: Papillary: 10; Chromophobe: 5; Sarcomatoid: 2; Collecting Duct: 2; Oncocytoma: 6	Clear cell: 542	Papillary: 291
Tumor size	< 4 cm: 19; ≥ 4 cm: 62; Unknown: 2	NA	NA
Grade	G0 (Oncocytoma): 6;<br>G1: 5; G2: 29; G3: 26; G4: 16; Unknown: 1	G1: 14; G2: 234;<br>G3: 208; G4: 78;<br>Unknown: 8	NA
Stage	pT0: 6; pT1a: 19 pT1b: 18; pT2: 12; pT3a: 11; pT3b: 15; pT4: 1; Unknown: 1	pT1: 165; pT1b: 113<br>pT2: 66; pT2b: 4<br>pT3: 127 pT3b: 56<br>pT4: 11	pT1a: 131; pT1b: 63; pT2a: 28; pT2b: 5; pT3: 47; pT3b: 12; pT4: 2; Unknown: 3
Lymph node invasion	(-): 51; (+): 5;<br>Unknown: 27	(-): 241; (+): 17;<br>Unknown: 284	
Lymphovascular invasion	(-): 43; (+): 7; Unknown: 33		
Karnofsky score	(+) 36; Unknown: 47<br>Mean: 86.4 ± 14.2; median: 90	(+): 28; Unknown: 514<br>Mean: 80.7 ± 34.6; median: 95	(+): 77; Unknown: 214<br>Mean: 87.7 ± 21.9; median: 90
Metastasis (clinical)<br>M-stage (TCGA)	(-): 64; (+): 18;<br>Unknown: 1	(-): 431; (+): 79<br>Unknown: 32	(-): 95; (+): 9; Unknown: 187
Follow-up (Metastasis):	33.1 ± 25.2 ; 27 months		
Overall survival		(-): 363; (+): 177;<br>Unknown: 2	(-): 244; (+): 44;<br>Unknown: 3
Follow-up (overall survival):		44.1 ± 32.2; 38.6 months	35 ± 29.6; 25.3 months

Supplementary Table 3: Relative intensities of the immunoblot data presented in various figures. For each sample the normalized value (Intensity of the protein of interest ÷ intensity of loading control) was obtained. The normalized value in a treated sample (e.g., SF+MU doses: 5/0.1, 5/0.2) was divided by the corresponding normalized value in the control sample (or Veh) to obtain the fold change value. Therefore, for the control, the fold change was equal to 1.

**Figure 1 B**	Samples	Fold changes
HAS3	HK-2	1
	786-O	22.52
	Caki-1	19.03
	769-P	21.59

**Figure 1E**	Samples SF μM		Fold changes
HAS3	786-O	0	1
		5	0.99
		10	0.39
		15	0.13
		20	0.01

**Figure 1F**	Samples	SFμM	Fold changes
HAS3	Caki-1	0	1
		5	0.91
		10	0.39
		15	0.18
		20	0.04

**Figure 2 C**	Samples		Fold changes
HAS3	NK	1	1
		2	0.77
		3	4.13
	Non-Met	1	2.83
		2	11.83
		3	10.31
	Met	1	24.63
		2	49.28
		3	26.02

Figure 3 C	Samples	SF µM	Fold changes
HAS3	786-O	C	1
		SF:5	0.97
		MU:0.1	0.88
		MU:0.2	0.86
		5/0.1	0.29
		5/0.2	0.04

Figure 3 D	Samples	SF µM	Fold changes
HAS3	Caki-1	C	1
		SF:5	0.63
		MU:0.1	0.85
		MU:0.2	0.65
		5/0.1	0.22
		5/0.2	0.26

Note: FLAG expression intensity fold changes (Figure 4A), are not shown as there is no FLAG-tag expression in the EV transfectants.

**Figure 4 B and C**						
		**786-O EV**			**786-O HAS3**	
**Protein**	C	5/0.1	5/0.2	C	5/0.1	5/0.2
HAS3	1	0.37	0.07	1	0.94	1.02
		**Caki-1 EV**			**Caki-1 HAS3**	
**Protein**	C	5/0.1	5/0.2	C	5/0.1	5/0.2
HAS3	1	0.64	0.15	1	1.05	1.29

**Figure 5 B**	Samples		Fold changes
HAS3	786-O	Ctrl shRNA	1
		HAS3 shRNA #1	0.21
		HAS3 shRNA #2	0.38
	Caki-1	Ctrl shRNA	1
		HAS3 shRNA #1	0.00
		HAS3 shRNA #2	0.01
**Note:** FLAG expression intensity fold changes (Supplementary Figure 2 A), are not shown as there is no FLAG-tag expression in the EV transfectants.			

Figure 6E (786-O) and 6F (Caki-1)						
		**786-O EV**			**786-O HAS3**	
**Protein**	C	5/0.1	5/0.2	C	5/0.1	5/0.2
Cyclin B1	1	0.54	3.72	1	1.03	1.16
Cyclin E1	1	0.07	0.07	1	0.90	1.07
p-CDK1	1	0.84	3.34	1	1.18	1.28
CDK1	1	1.25	0.96	1	1.06	1.18
p-Rb	1	0.64	0.46	1	1.08	1.17
Rb	1	0.92	0.61	1	0.94	1.01
Mcl-1	1	0.18	0.14	1	0.88	0.59
Cl. Cas-3	1	2.75	6.97	1	1.02	0.97
Cl. PARP	1	0.89	0.51	1	0.89	0.78
		**Caki-1 EV**			**Caki-1 HAS3**	
**Protein**	C	5/0.1	5/0.2	C	5/0.1	5/0.2
Cyclin D1	1	0.74	0.14	1	0.73	0.82
Cyclin E1	1	0.63	0.49	1	1.06	0.94
p-CDK2	1	0.71	0.52	1	1.37	1.37
CDK2	1	1.65	1.42	1	1.43	0.85
p21	1	7.64	8.19	1	1.93	1.36
p-Rb	1	1.03	0.12	1	1.21	1.07
Rb	1	0.97	0.58	1	1.31	1.00
Mcl-1	1	1.09	0.13	1	0.95	1.27
Cl. Cas-3	1	19.23	34.75	1	1.16	1.09
Cl. PARP	1	3.98	6.07	1	0.61	0.75

Figure 7 B						
		**786-O EV**			**786-O HAS3**	
**Protein**	C	5/0.1	5/0.2	C	5/0.1	5/0.2
p-MET	1	0.16	0.08	1	0.89	0.82
MET	1	1.37	1.25	1	0.80	0.93
CD44	1	0.89	0.43	1	0.97	0.95
RHAMM	1	0.04	0.06	1	1.22	1.20
MMP-9	1	0.12	0.12	1	0.70	0.84
Caveolin-1	1	0.23	0.17	1	1.06	0.94
		**Caki-1 EV**			**Caki-1 HAS3**	
**Protein**	C	5/0.1	5/0.2	0	5/0.1	5/0.2
pMET	1	0.59	0.27	1	0.83	0.75
MET	1	0.88	0.78	1	0.82	0.83
CD44	1	0.76	0.56	1	1.02	0.83
RHAMM	1	0.33	0.01	1	1.13	0.83
MMP-9	1	0.05	0.03	1	0.91	0.82
Caveolin-1	1	0.40	0.12	1	0.88	0.59

**Figure 8D**								
	**EV**				**HAS3**			
Protein	**Veh #1**	**Veh #2**	**SF+MU #1**	**SF+MU #2**	**Veh #1**	**Veh #2**	**SF+MU #1**	**SF+MU #2**
CD44	1	2.07	0.01	0.01	1	0.78	0.70	0.71
p-MET	1	0.62	0	0	1	0.75	1.37	0.93
MET	1	0.70	0.91	1.21	1	1.02	1.12	1.23
p-c-RAF	1	1.39	0.41	0.27	1	0.93	1.35	1.18
RAF	1	0.97	0.66	0.95	1	0.78	0.58	0.68

Supplementary Table 4: Multivariate analysis to determine the relationship of clinical parameters and HAS3 levels to metastasis and OS. Cox Proportional Hazards Model was used to evaluate the ability of clinical parameters and HAS3 expression to associate with metastasis in the clinical cohort and with OS in TCGA cohort. Parameters included: Age, sex, tumor size, T-stage, low/high grade, lymphovascular invasion, renal vein invasion, HAS3. Renal vein invasion and tumor size data were not available in TCGA dataset, but M-stage was included. Only the parameters that reached significance are shown.

	Clinical specimen cohort		
	Metastasis		
Parameter	χ^2	P-value	Range Hazard Ratio, 95% CI
T-stage	6.05	0.0139	4.64; 0.27-81.11
HAS3	6.89	0.0087	1.21; 1.05-1.43
	TCGA-KIRC cohort		
	χ^2	P-value	Range Hazard Ratio, 95% CI
Age	8.80	0.0030	1.03; 1.01-1.05
M-stage	15.87	<0.0001	2.83; 1.70-4.72
	TGA-KIRP cohort		
T-stage	8.31	0.040	3.168;
N-stage	6.86	0.0088	4.03

Appendix – Uncropped Images of the blots presented in the indicated figures.

Raw Data

Figure 1B

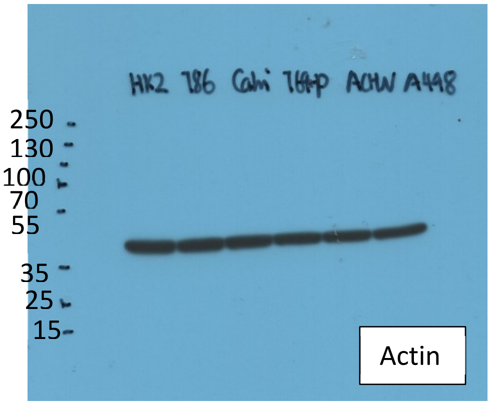

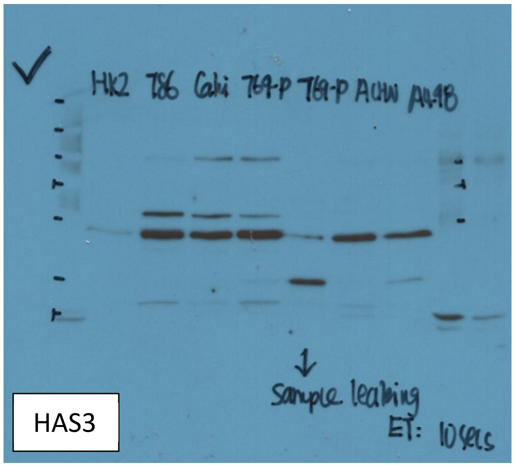

Figure 1E

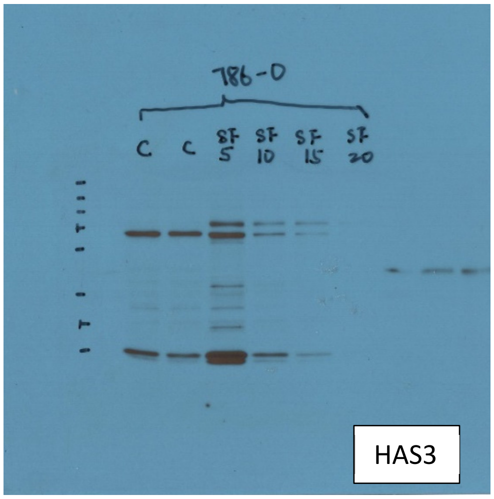

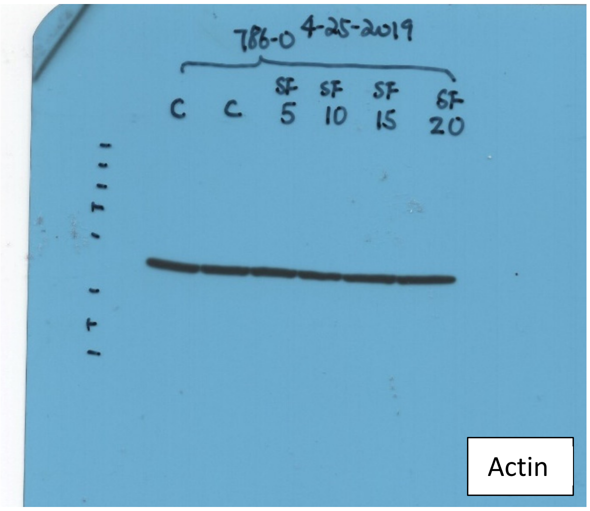

Figure 1F

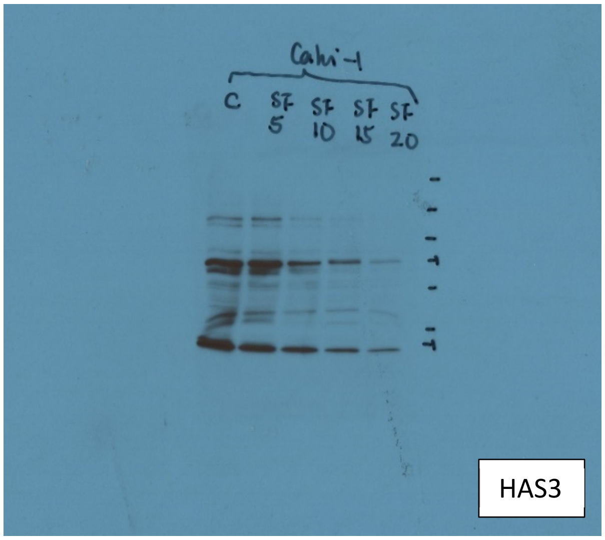

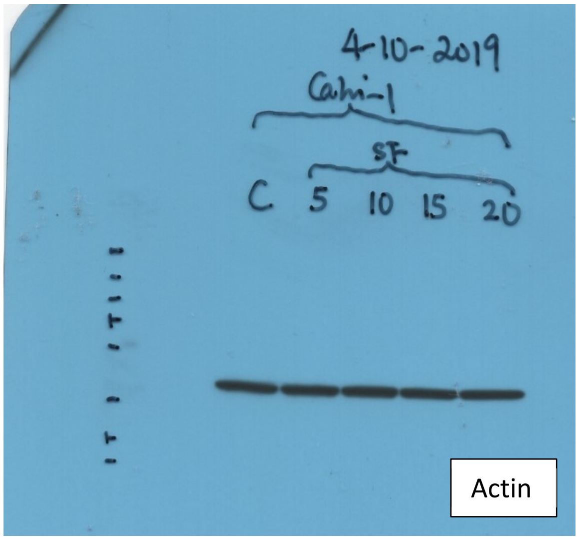

Figure 2C

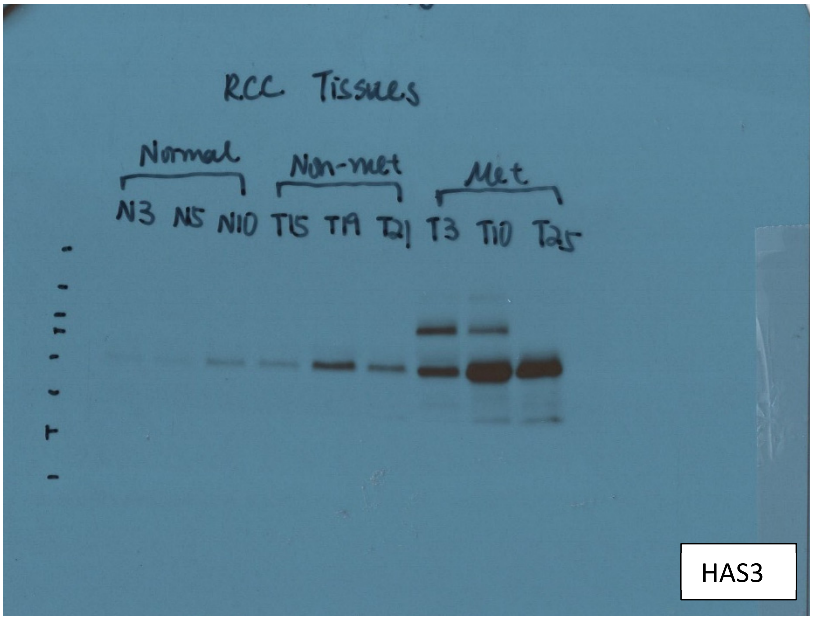
RCC Tissues
Normal
Non-met
Met
N3 N5 N10 T15 T19 T21 T3 T10 T25
HAS3

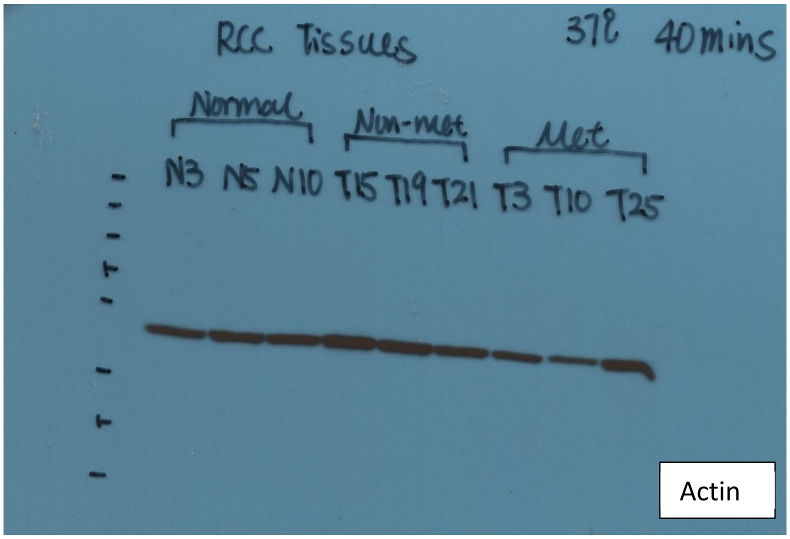
RCC Tissues
378 40mins
Normal
Non-met
Met
N3 N5 N10 T15 T19 T21 T3 T10 T25
Actin

Figure 3C

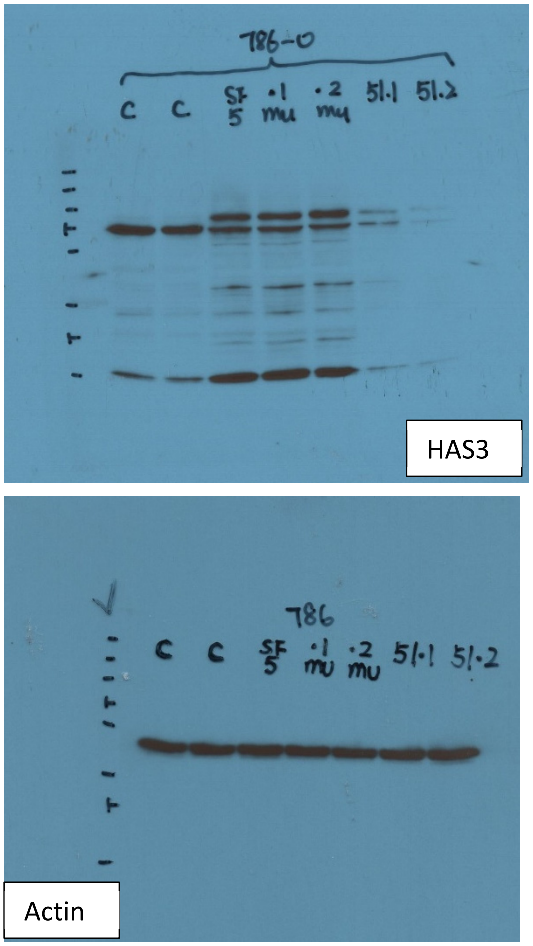

Figure 3D

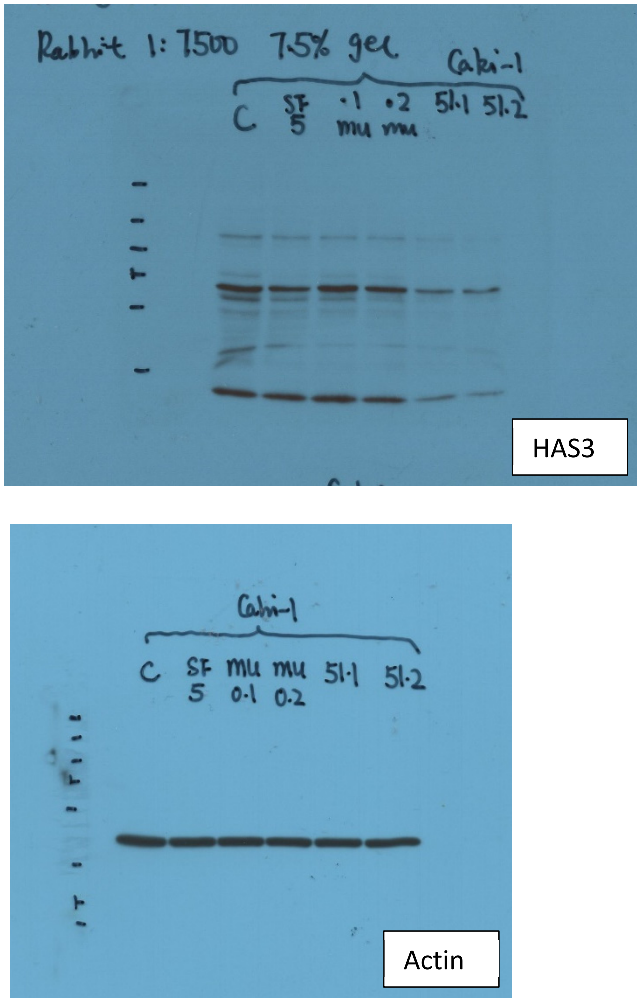

Figure 4 A

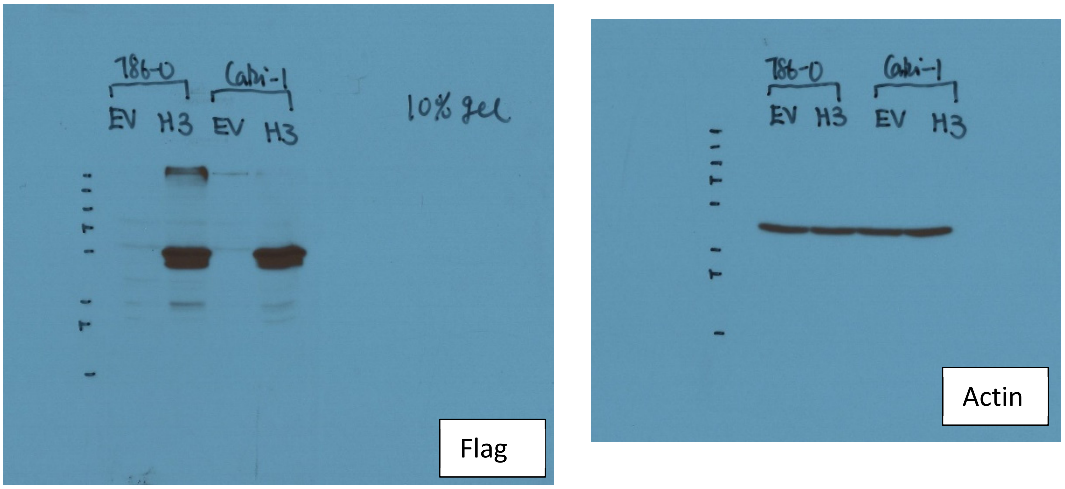

Figure 4B

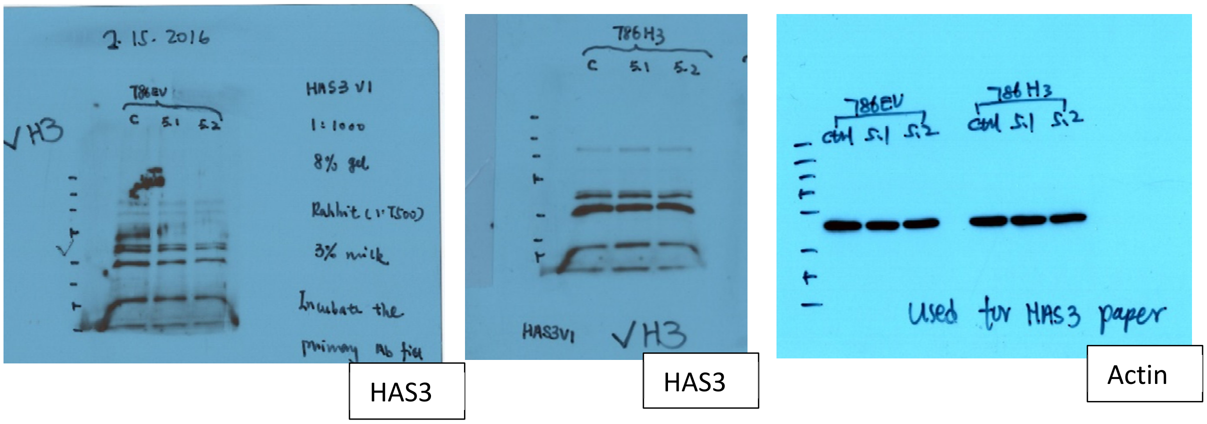

Figure 4C

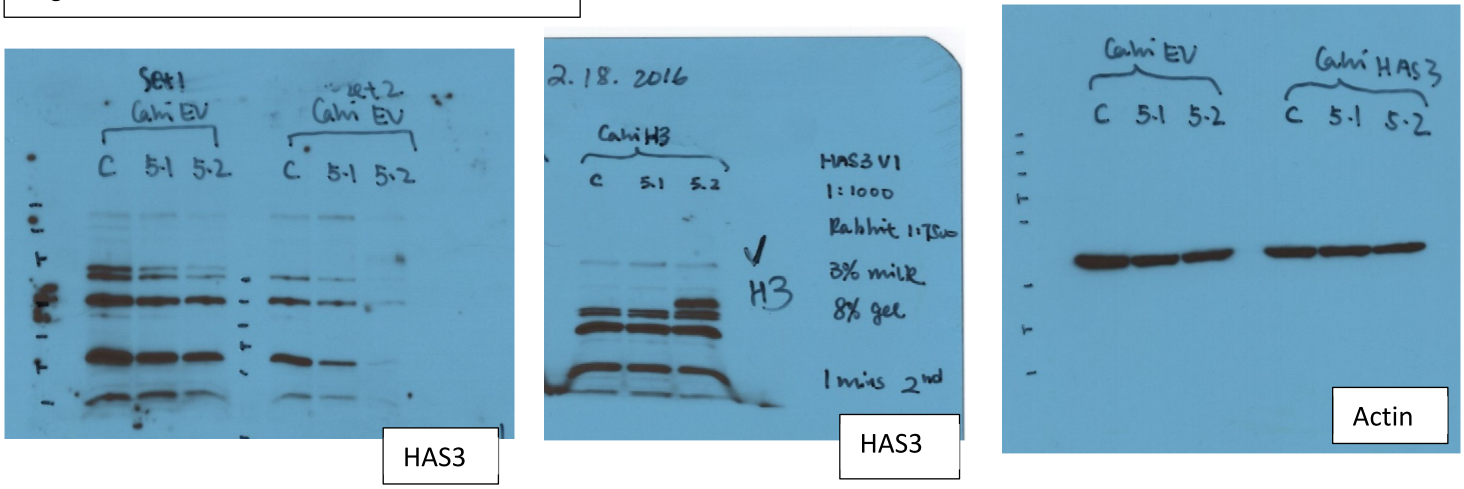

Figure 5B

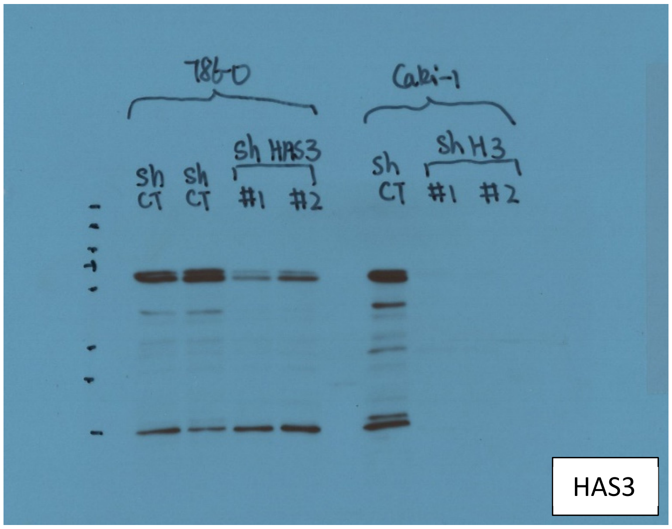
786-O
Caki-1
sh CT
sh CT
sh HAS3
#1
#2
sh CT
sh H3
#1
#2
HAS3

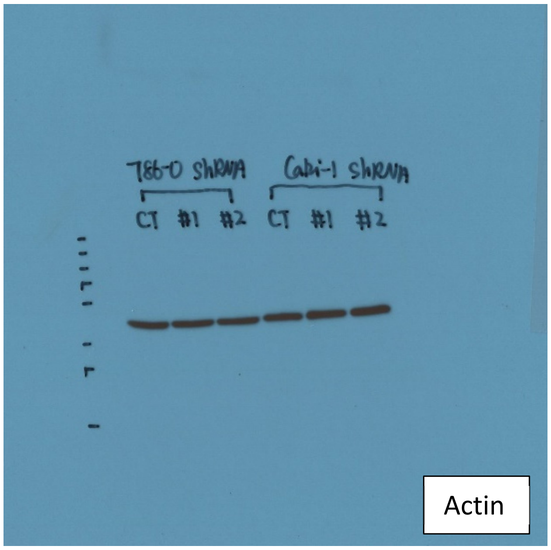
786-O shRNA
Caki-1 shRNA
CT
#1
#2
CT
#1
#2
Actin

Figure 6E & F

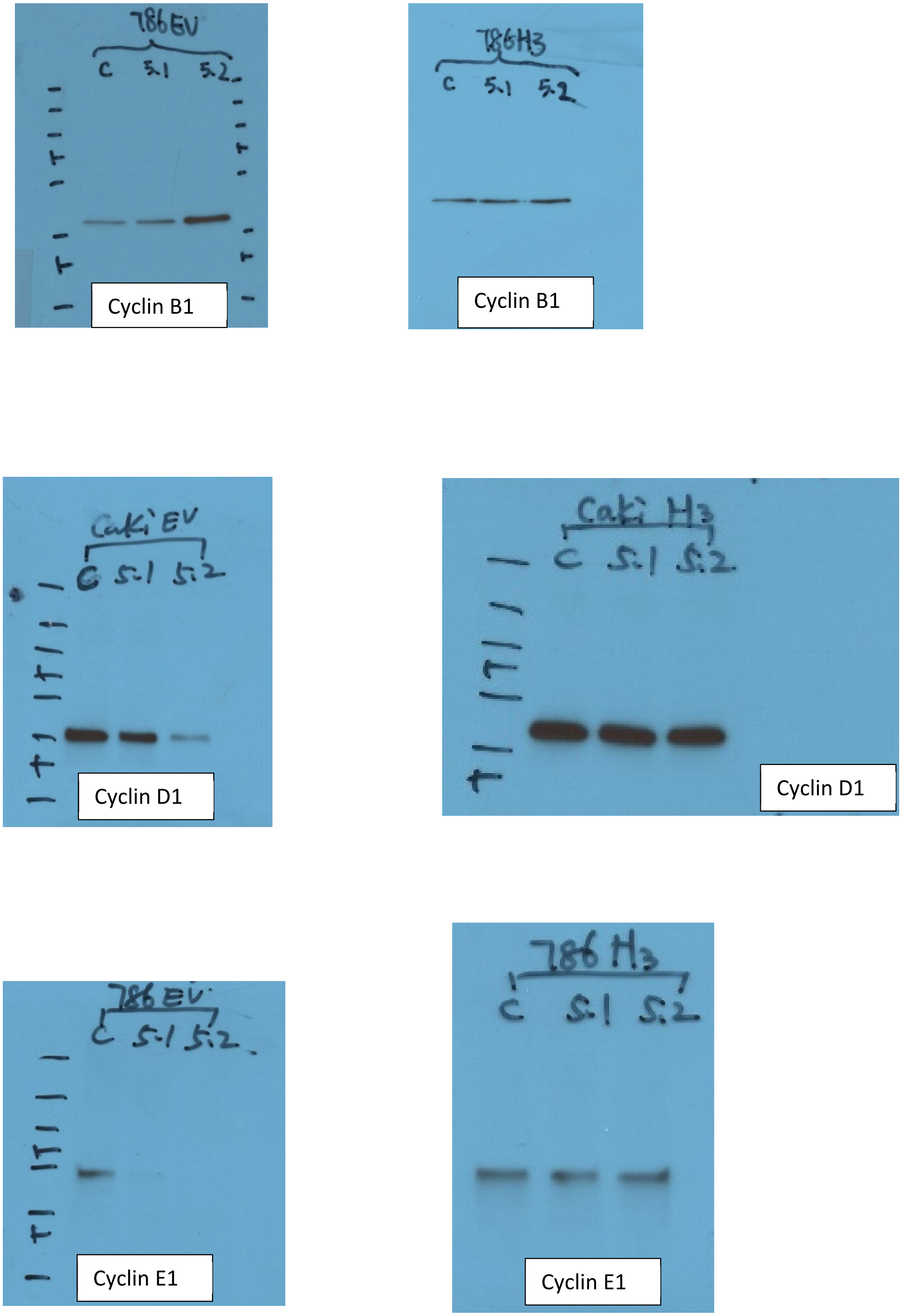
786EV
C 5.1 5.2
Cyclin B1
786H3
C 5.1 5.2
Cyclin B1
Caki EV
C 5.1 5.2
Cyclin D1
Caki H3
C 5.1 5.2
Cyclin D1
786EV
C 5.1 5.2
Cyclin E1
786 H3
C 5.1 5.2
Cyclin E1

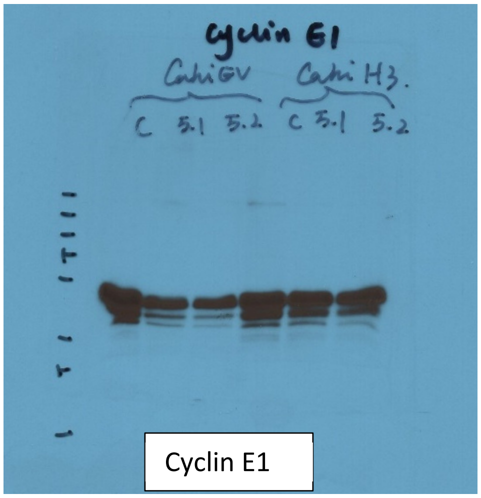

Cyclin E1

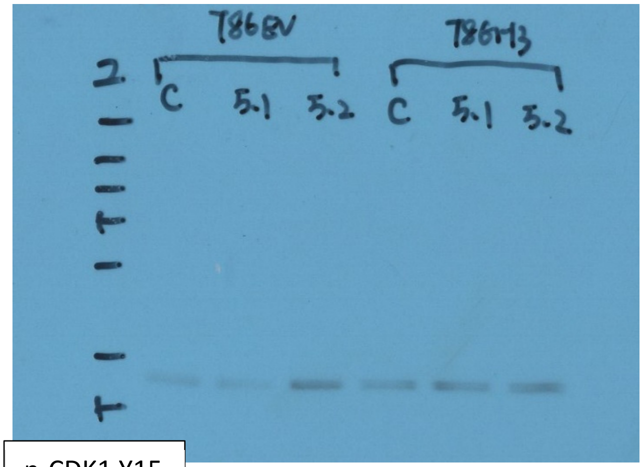

p-CDK1 Y15

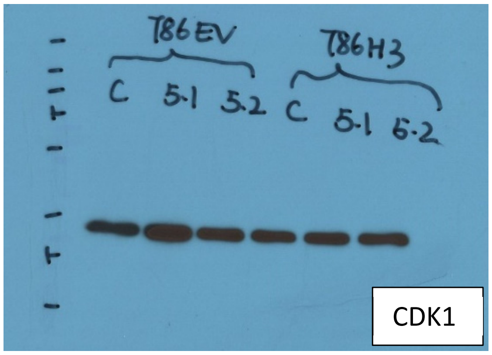

CDK1

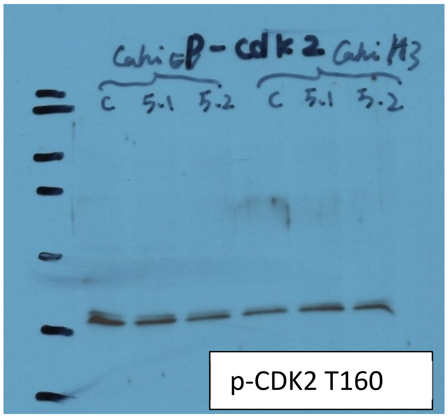

p-CDK2 T160

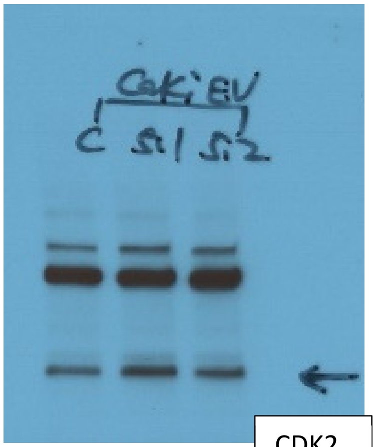
CDK2

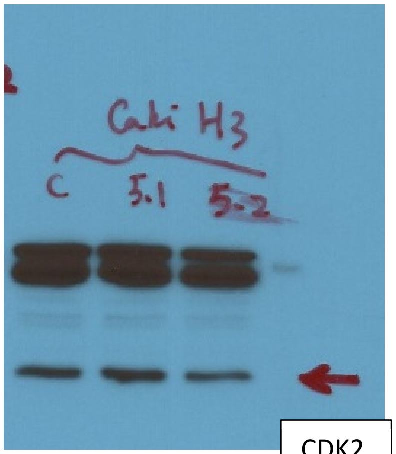
CDK2

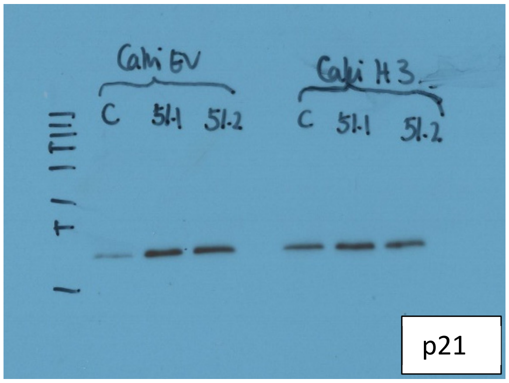
Caki EV
Caki H3
C 51.1 51.2
C 51.1 51.2
p21

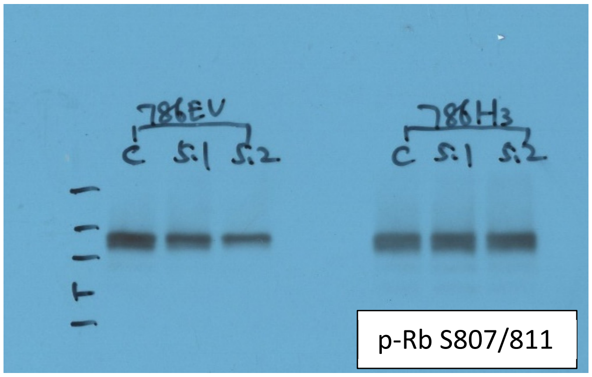
786EV
786H3
C S.1 S.2
C S.1 S.2
p-Rb S807/811

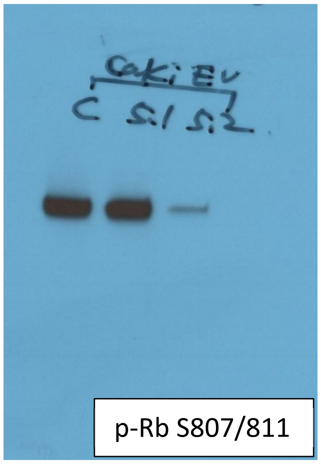
Caki EV
C S.1 S.2
p-Rb S807/811

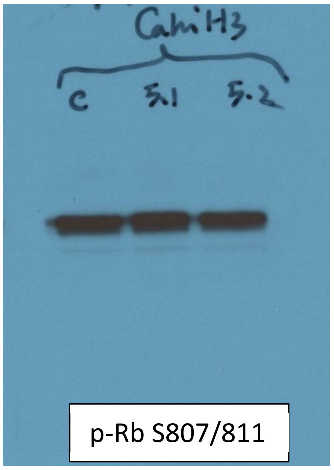
Caki H3
C S.1 S.2
p-Rb S807/811

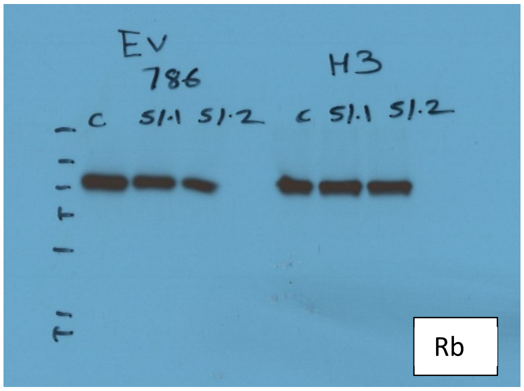
EV
786
H3
C 51.1 51.2
C 51.1 51.2
Rb

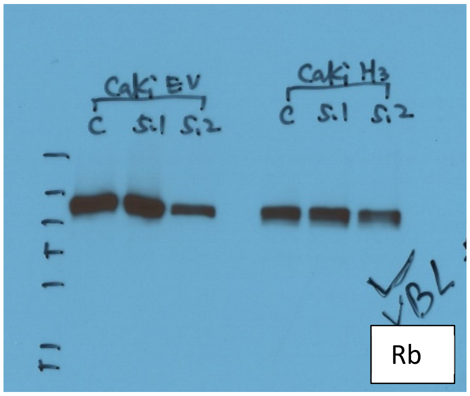
Caki EV
Caki H3
C S.1 S.2
C S.1 S.2
Rb

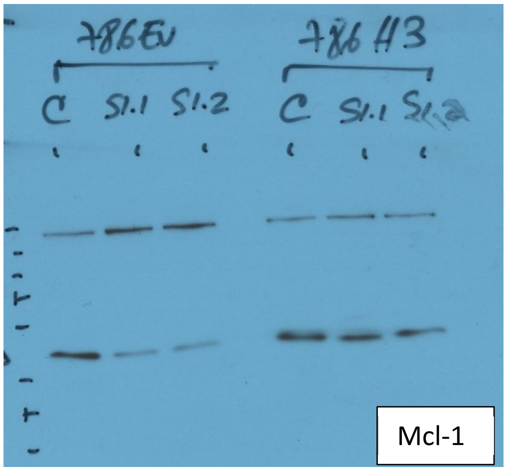

Mcl-1

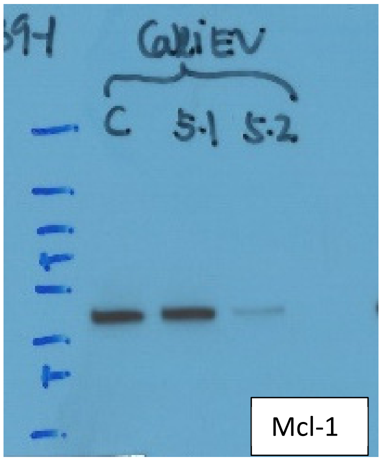

Mcl-1

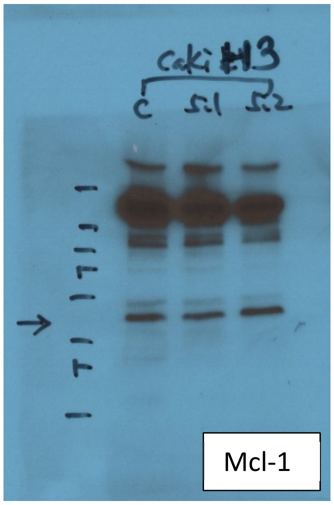

Mcl-1

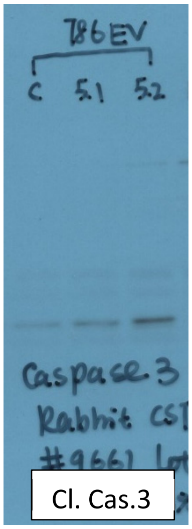

Cl. Cas.3

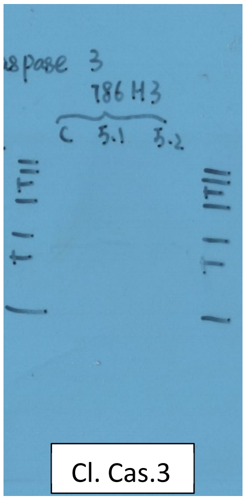

Cl. Cas.3

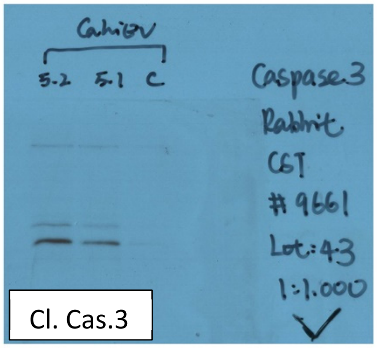

Cl. Cas.3

Note the order in Caki EV

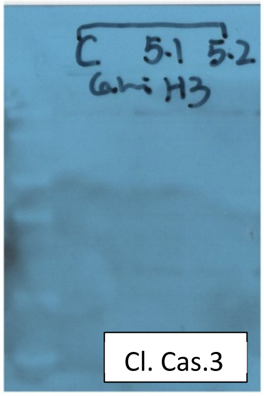

Cl. Cas.3

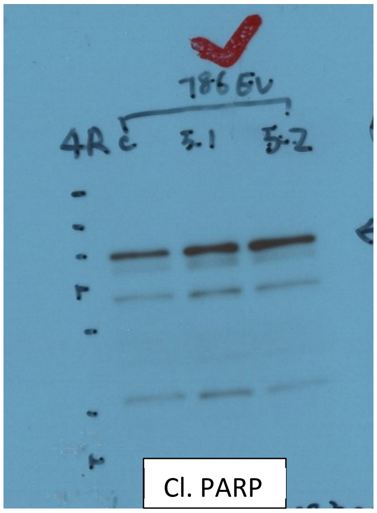

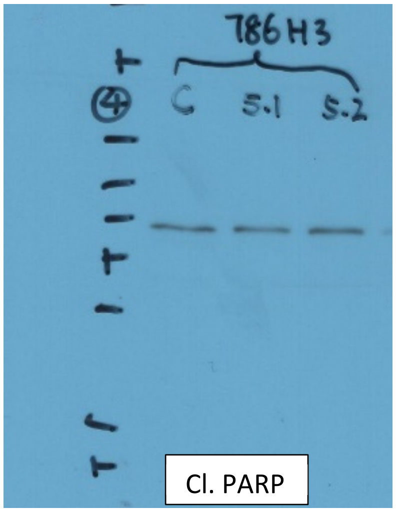

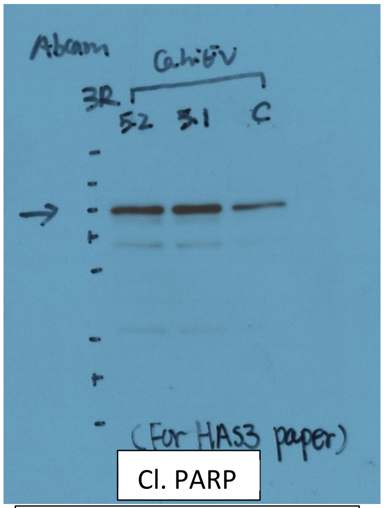

Note the order in Caki EV

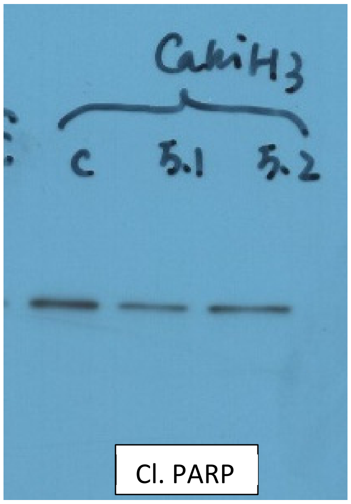

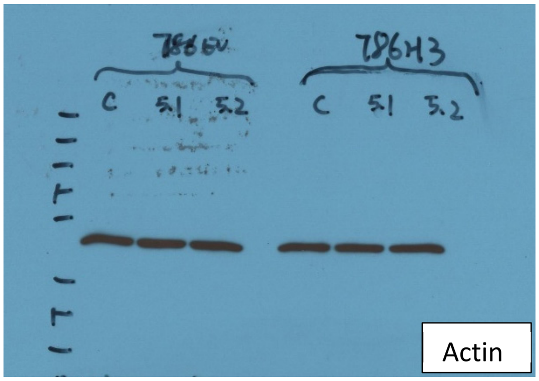

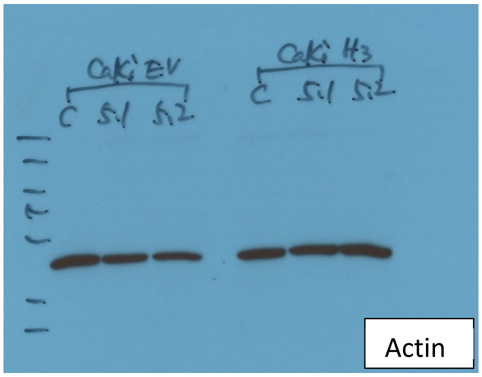

Figure 7B

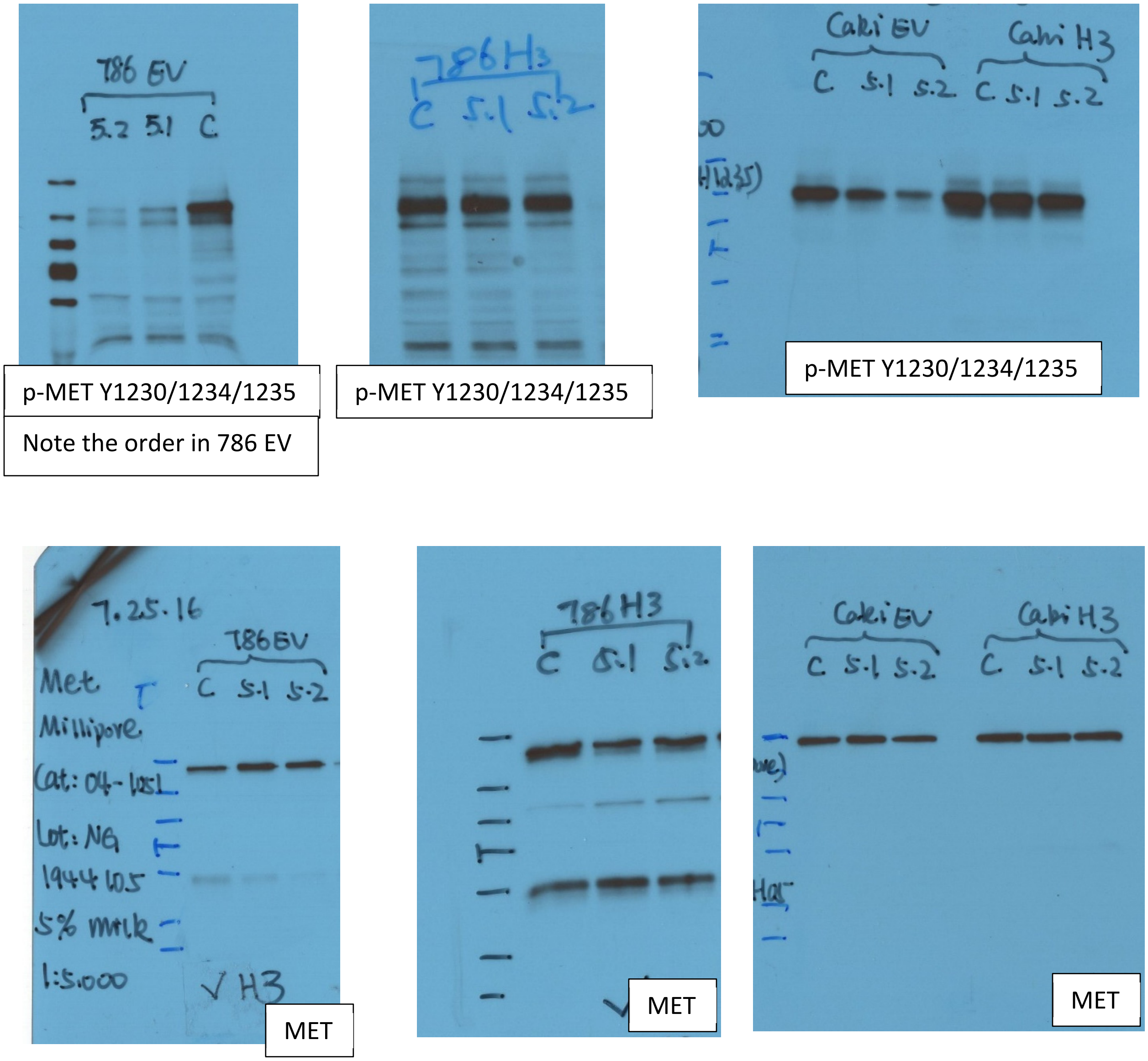
786 EV
5.2 5.1 C
p-MET Y1230/1234/1235
Note the order in 786 EV
786 H3
C 5.1 5.2
p-MET Y1230/1234/1235
Caki EV
C 5.1 5.2
Caki H3
C 5.1 5.2
p-MET Y1230/1234/1235
7.25.16
786 EV
C 5.1 5.2
Met
Millipore
Cat: 04-1051
Lot: NG
1944105
5% milk
1:5,000
√ H3
MET
786 H3
C 5.1 5.2
MET
Caki EV
C 5.1 5.2
Caki H3
C 5.1 5.2
MET

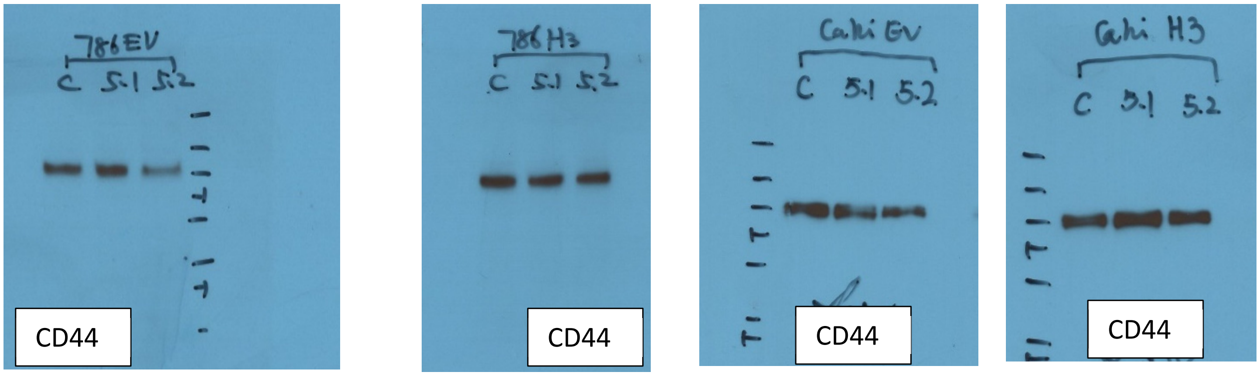
786EV
C 5.1 5.2
CD44
786H3
C 5.1 5.2
CD44
Caki EV
C 5.1 5.2
CD44
Caki H3
C 5.1 5.2
CD44

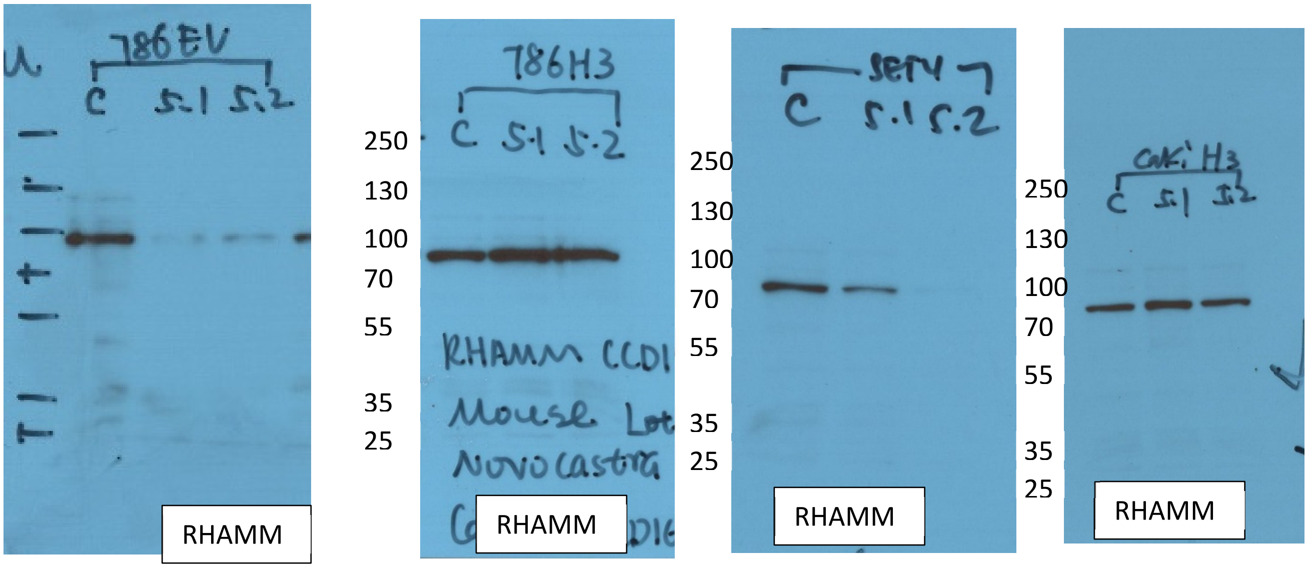
786EV
C 5.1 5.2
RHAMM
250
130
100
70
55
35
25
786H3
C 5.1 5.2
RHAMM
250
130
100
70
55
35
25
C 5.1 5.2
RHAMM
250
130
100
70
55
35
25
Caki H3
C 5.1 5.2
RHAMM

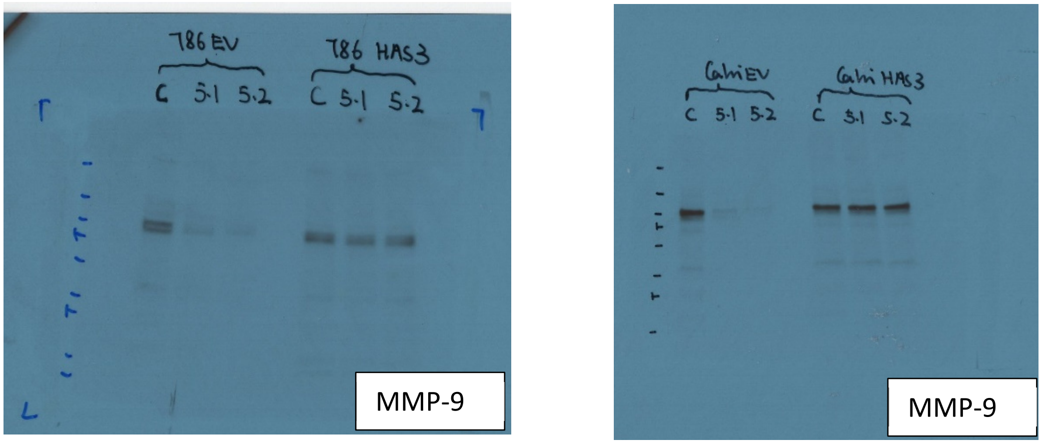
786 EV
C 5.1 5.2
786 HAS3
C 5.1 5.2
MMP-9
Caki EV
C 5.1 5.2
Caki HAS3
C 5.1 5.2
MMP-9

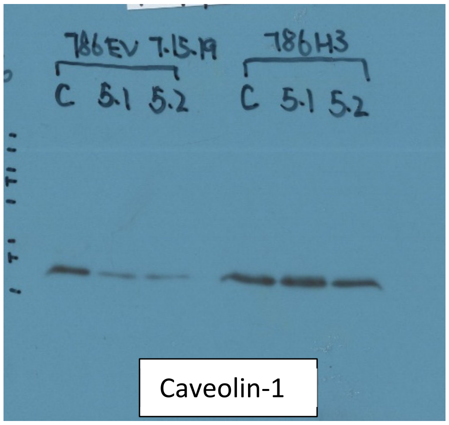
786EV 7.15.19
786H3
C 5.1 5.2
C 5.1 5.2
Caveolin-1

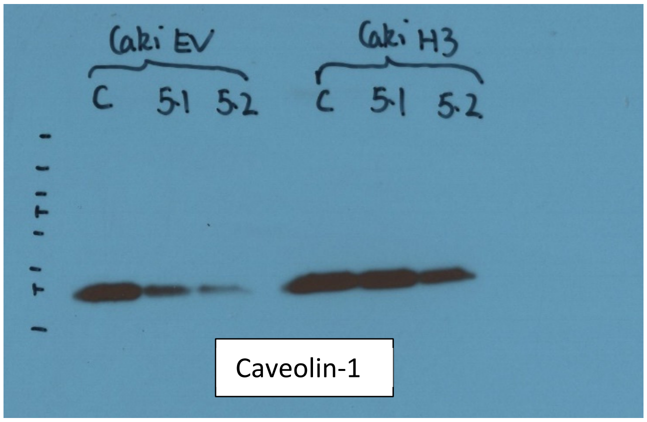
Caki EV
Caki H3
C 5.1 5.2
C 5.1 5.2
Caveolin-1

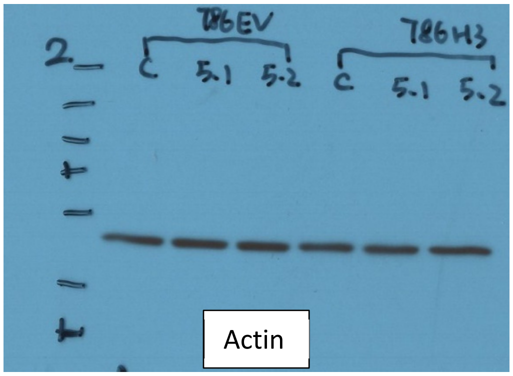
786EV
786H3
C 5.1 5.2
C 5.1 5.2
Actin

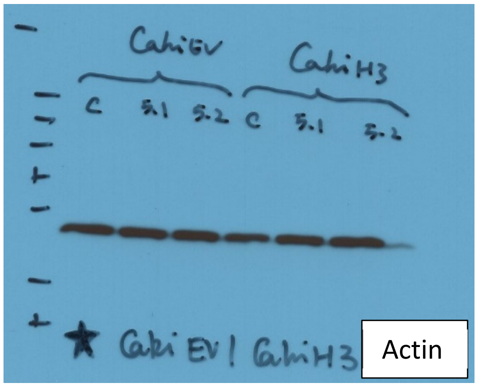
CakiEV
CakiH3
C 5.1 5.2
C 5.1 5.2
Caki EV / CakiH3
Actin

Figure 8D

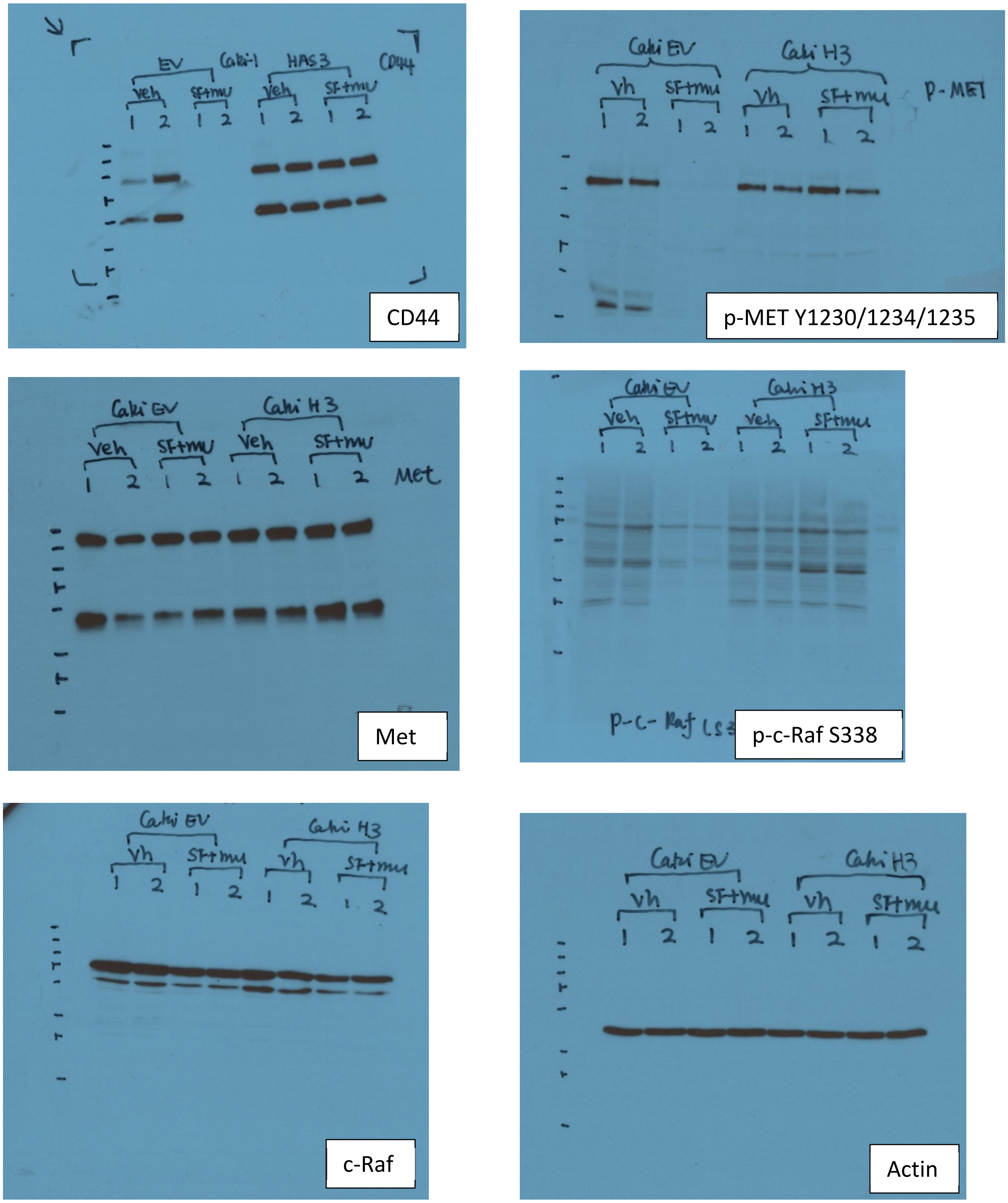

Supplement: Supplementary file 1 — Additional file 1: Table S1. Materials used in this study. Description of antibodies, reagents and primers used in this study. Table S2. Characteristics of clinical and TCGA RCC cohorts. Clinical cohort consists of 129 specimens acquired from 83 RCC patients (46 normal; 83 tumor). TCGA-KIRC and TCGA-KIRP datasets were downloaded from UCSC Xena (Xena functional explorer) Mean ± SD and median are reported. OS: (−) designates survival, (+) designates death. Table S3. Relative intensities of the immunoblot data presented in various figures. For each sample the normalized value (Intensity of the protein of interest ÷ intensity of loading control) was obtained. The normalized value in a treated sample (e.g., SF+MU doses: 5/0.1, 5/0.2) was divided by the corresponding normalized value in the control sample (or Veh) to obtain the fold change value. Therefore, for the control, the fold change was equal to 1. Table S4. Multivariate analysis to determine the relationship of clinical parameters and HAS3 levels to metastasis and OS. Cox Proportional Hazards Model was used to evaluate the ability of clinical parameters and HAS3 expression to associate with metastasis in the clinical cohort and with OS in TCGA cohort. Parameters included: Age, sex, tumor size, T-stage, low/high grade, lymphovascular invasion, renal vein invasion, HAS3. Renal vein invasion and tumor size data were not available in TCGA dataset, but M-stage was included. Only the parameters that reached significance are shown. [file 12935_2022_2818_MOESM1_ESM.pdf]
